# MicroRNA-223 Induced Repolarization of Peritoneal Macrophages Using CD44 Targeting Hyaluronic Acid Nanoparticles for Anti-Inflammatory Effects

**DOI:** 10.1371/journal.pone.0152024

**Published:** 2016-05-05

**Authors:** Thanh-Huyen Tran, Swathi Krishnan, Mansoor M. Amiji

**Affiliations:** Department of Pharmaceutical Sciences, School of Pharmacy, Bouve College of Health Sciences, Northeastern University, 360 Huntington Avenue, Boston, MA, 02115, United States of America; Universidad de Castilla-La Mancha, SPAIN

## Abstract

The aim of this study was to evaluate macrophages repolarization from pro-inflammatory M1 to anti-inflammatory M2 phenotype upon transfection with microRNA-223 (miR-223) duplexes and miR-223 expressing plasmid DNA encapsulated in CD44-targeting hyaluronic acid-poly(ethyleneimine) (HA-PEI) nanoparticles (NPs). The HA-PEI/miR-223 NPs with spherical shape and an average diameter of 200 nm were efficiently internalized by J774A.1 alveolar and primary peritoneal macrophages and non-cytotoxic at HA-PEI concentration less than 200 μg/mL. Transfection of HA-PEI/miR-223 NPs in J774A.1 macrophages showed significantly higher miR-223 expression than that with HA-PEI/plasmid DNA expressing miR-223 (pDNA-miR-223). HA-PEI/miR-223 NPs mediated transfection increased miR-223 expression to 90 fold in primary peritoneal macrophages compared to untreated cells. The overexpression of miR-223 in both J774A.1 and peritoneal macrophages induced a phenotypic change from M1 to M2 state as indicated by a decrease in iNOS-2 (M1 marker) and an increase in Arg-1 (M2 marker) levels compared to those in lipopolysaccharide (LPS) and interferon-gamma (IFN-γ)-stimulated macrophages (M1). The change in macrophage phenotype by HA-PEI/miR-223 NPs could suppress the inflammation in peritoneal macrophages induced by LPS as evidenced by a significant decrease in pro-inflammatory cytokine levels TNF-α, IL-1β and IL-6, compared to LPS-stimulated peritoneal macrophages without treatment. The results demonstrated that miR-223-encapsulated HA-PEI NPs modulated macrophage polarity toward an anti-inflammatory M2 phenotype, which has potential for the treatment of inflammatory diseases.

## Introduction

Tissue-associated macrophages, derived from myeloid precursor cells in the bone marrow, are important cellular components of the host innate and adaptive immune system. Macrophages are highly plastic cells, which are maintained under physiological homeostasis in a spectrum of functional polarization states based on the microenvironmental signaling [1,2]. Contemporary evidence suggests that macrophages are maintained in a spectrum of pro-inflammatory M1 phenotype or anti-inflammatory M2 phenotype depending on the local environmental stimuli. In the presence of an inflammatory stimulus, such as with interferon gamma (IFN-γ) and lipopolysaccharide (LPS), macrophages specifically polarize to M1 phenotype with increased production of pro-inflammatory cytokines such as tumor necrosis factor alpha (TNF-α), interleukin-1beta (IL-1β) and interleukin-6 (IL-6) [3]. On the other hand, in the wound-healing environment or in the presence of anti-inflammatory cytokines, such as IL-4 or IL-10, macrophages shift to a predominant M2 phenotype with high secretion of anti-inflammatory cytokines IL-4, IL-10, transforming growth factor beta (TGF-β) for tissue repair and re-modeling [4,5]. It has been reported that macrophages exist predominantly in M1 phenotype causing tissue damage in chronic inflammatory and autoimmune diseases [6]. Therefore, a strategic approach that can switch macrophage phenotype from an M1 to M2 state may be a promising modality for the treatment of chronic inflammatory diseases. However, there has been no therapy for macrophage-targeting and modulating their phenotype currently on the market, which open a new area for investigation [2].

MicroRNAs (miRNAs) are small and non-coding RNAs of 18–25 nucleotides that regulate the expression of multiple protein-encoding genes at the post-transcriptional level by inhibiting translation or inducing mRNA degradation [7,8]. Recently, miRNAs have emerged as novel molecular regulators of numerous genes and pathways involved in normal immune responses, in the pathogenesis of cancers, inflammatory and autoimmune diseases [3]. In addition, miRNAs have been reported to regulate macrophage polarization and function. Among the known miRNAs, miR-223 is a potent regulator of inflammatory responses. miR-223 is highly enriched in bone marrow-derived macrophages and macrophages isolated from adipose tissue [9]. *In vitro*, miR-223–deficient macrophages highly respond to LPS through the induced expression of pro-inflammatory cytokines, such as TNF-α, IL-6, and IL-1β. In contrast, the expression of arginase-1 (Arg1), a marker for M2 macrophages, was blunted in the absence of miR-223 [9]. Furthermore, depletion of miR-223 significantly promotes macrophage infiltration and M1 macrophage activation, which subsequently elevates the state of inflammation in adipose tissues and exacerbates insulin resistance in the diet-induced obese mouse model [9]. The study indicated a potential role of miR-223 in inflammation via macrophage function regulation. However, the role of miR-223 overexpression in macrophages for anti-inflammatory therapy has not been investigated yet.

The biggest challenge for nucleic acid (e.g., miRNA)-based systemic therapy is the poor efficiency of delivery to the target tissue or cell. The unprotected miRNA cargo undergo rapid degradation in the circulation by the serum nucleases [10]. Furthermore, miRNAs or other nucleic acids have poor cell membrane permeability due to their high molecular weight, hydrophilic property, and negative charge [11], and these factors represent a major challenge for efficient cellular uptake. Once taken up by the cells, the endosome entrapment limits the release of miRNAs to cytoplasm, which further reduces their activity. Therefore, there is a critical need for a delivery system that effectively ensures miRs bioavailability to macrophages upon local or systemic administration. Hyaluronic acid-poly(ethylene imine) (HA-PEI) conjugate has emerged as an ideal delivery system for nucleic acids, due to the biodegradable, biocompatible, non-toxic, non-immunogenic properties of HA and PEI’s high capacity for nucleic acid complexation [12–17]. In addition, HA has been reported to specifically bind to CD44 receptors overexpressed on macrophages [12,13]. Furthermore, PEI has ability to facilitate endosomal escape via “proton sponge effect” for improved cytosolic delivery [18]. Our previous studies, we have demonstrated successful delivery of siRNA by HA-PEI NPs to tumor tissues for anti-cancer therapy [13,16,17]. In this study, HA-PEI conjugate was synthesized for encapsulation and specific delivery of miR-223 duplex to peritoneal macrophages for modulation of their functional polarity toward anti-inflammatory M2 phenotypes as a platform for the treatment of inflammatory diseases by miRNA therapy.

## Materials and Methods

### Materials

Sodium hyaluronate (HA, 20,000 Da) was purchased from Lifecore Biomedical Co. (Chaska, MN). Branched poly(ethyleneimine) (bPEI, 10,000 Da) was obtained from Polysciences Inc. (Warrington, PA). N-(3-dimethylaminopropyl)-N’-ethylcarbodiimide hydrochloride (EDC), N-hydroxysuccinimide (NHS), Brewer-thioglycollate medium, and lipopolysaccharide (LPS) were purchased from Sigma-Aldrich Chemical Co. (St. Louis, MO, USA). Dulbecco’s modified Eagle medium (DMEM) was purchased from Cellgro (Manassas, VA) and fetal bovine serum (FBS) was obtained from HyClone (Logan, UT). Murine IFN-γ was obtained from PeproTech (Rocky Hill, NJ). 4’, 6-diamidino-2-phenylindole (DAPI) were purchased from Invivogen (San Diego, CA). miR-223 mimic, Taqman^®^ probes specific for miR-223, 4% agarose gel, nuclease-free water, and Lipofectamine^®^ transfection iMAX, and penicillin/streptomycin antibiotics were purchased from Life Technologies (Woburn, MA). *E*. *coli* transformed with plasmid DNA expressing mouse miR-223 (pEGP-mmu-miR-223) was purchased from Cell Biolabs (San Diego, CA). The plasmid was then amplified in a bacterial culture containing ampicillin, followed by isolation and purification using a Plasmid Mega Kit following the manufacturer’s instructions. Cy5-NHS ester was obtained from Lumiprobe (Hallandale Beach, FL). Primers specific for iNOS-2, TNF-α, Arg-1, IL-1β, IL-6 and β-actin were purchased from Eurofins MWG Operon (Huntsville, AL).

### Synthesis of HA-PEI conjugate, preparation and characterization of HA-PEI/miRNA NPs

HA-PEI conjugate was synthesized by coupling reaction between HA and PEI in the presence of EDC/NHS. Briefly, HA (100 mg) and PEI (15 mg) were dissolved in distilled water (10 mL) containing NaCl (0.5 M). A solution containing EDC (35 mg) and NHS (22 mg) dissolved in distilled water was then added to the polymer mixture. The reaction mixture was stirred at room temperature for 24 h and then dialyzed against distilled water using a dialysis membrane (MWCO: 25 kDa) for 24 h. The HA-PEI conjugate was obtained after lyophilization. The lyophilized HA-PEI conjugate dissolved in D_2_O containing sodium chloride was characterized by 500 MHz ^1^H NMR spectroscopy (Varian Inc., CA).

HA-PEI/miR-223 nanoparticles (NPs) were prepared by mixing the HA-PEI solution in phosphate buffer saline (PBS, pH 7.4) with miR-223 mimic at an appropriate ratio (w/w) using a vortex mixer and incubating the complex for 10 min at room temperature.

The average particle size, size distribution and zeta-potential of HA-PEI/miR-223 NPs were measured using a dynamic light scattering (DLS) instrument (Malvern Zetasizer, Westborough, MA). The morphology of the NPs was imaged by transmission electron microscopy (TEM) (JEOL, Peabody, MA). Specimens were prepared by adding a suspension of the nanoparticles drop-wise to a Formvar/carbon film grid followed by air-drying.

To determine the optimal ratio of HA-PEI to miR-223 for the formation of HA-PEI/miR-223 NPs, different NPs were prepared with different HA-PEI to pDNA ratios (w/w) of 27:1, and 9:1. The NPs were then run on 4% agarose gel to check percent encapsulation of the miRNA. miRNA encapsulation was further confirmed by decomplexing HA-PEI/miR-223 NPs with anionic polyacrylic acid (PAA) by mixing an equal volume of HA-PEI/miRNA-223 and PAA using a vortex mixer. The strongly anionic PAA displaces the miRNA by electrostatically interacting with the cationic PEI. The decomplexed samples were then run on a 4% agarose gel to ensure the presence of intact miRNA bands.

### MTT assay for cytotoxicity of HA-PEI NPs

J774A.1 adherent murine macrophages (7500 cells/well) were seeded on 96-well plates and cultured in 200 μL of DMEM supplemented with 10% FBS, and 1% antibiotics for 24 h at 37°C and 5% CO2. After incubation, various concentrations of blank HA-PEI NPs (1 μg−1000 μg/mL) dissolved in DMEM without supplements were added. After 24 h of incubation, cytotoxicity was determined using 3-[4,5-dimethylthiazol-2-yl]-3,5-diphenyltetrazolium bromide dye (MTT dye, final concentration of 0.5 mg/mL) uptake at 570 nm on BioTek Synergy HT microplate reader.

### Isolation of peritoneal macrophages from C57BL/6 mice

All animal studies were performed according to an approved protocol by Institutional Animal Care and Use Committee (IACUC) at Northeastern University. C57BL/6 mice (8–10 weeks old) were purchased from Harlan Laboratories (South Easton, MA). After acclimation for up to 48 h, the mice were injected intraperitoneally with sterile Brewer-thioglycollate medium (2 mL, 4% w/v) to recruit macrophages to peritoneal cavity. Four days post-injection, the mice were sacrificed for macrophage collection from mouse peritoneal cavity using cold PBS. The cell pellets was obtained after centrifugation at 2,000 rpm for 5 min. The cells will be incubated in DMEM supplemented with 10% FBS and 1% antibiotics for 2 h and washed three times to remove non-adherent cells. The cells were cultured for uptake, transfection, polarization and anti-inflammatory studies.

### Intracellular uptake of HA-PEI/miRNA in macrophages

To observe the cellular uptake, HA-PEI was conjugated with a red fluorescence dye, Cy5. Peritoneal macrophages and J774A.1 macrophages were seeded at a density of 1.0 × 10^5^ cells/well in an 8-well Lab-Tek II chamber slide and pre-incubated for 24 h at 37°C and 5% CO_2_. Serum-free DMEM containing HA-PEI/miRNA NPs at an equivalent dose of 100 nM of miRNA was added to each well, followed by incubation for 2 h at 37°C. After incubation, the cells were washed twice with PBS and fixed in formalin (4%) for 15 min at room temperature, followed by nucleus staining with 1.0 μg/mL of DAPI. Cover glasses were then placed on glass slides. The cellular uptake of HA-PEI/miRNA NPs was imaged on a Zeiss confocal microscope (Carl Zeiss, Cambridge, UK) at an excitation wavelength of 647 nm for Cy5.

### *In vitro* transfection studies

Transfection study of miR-223 duplexes and plasmid DNA expressing miR-223 (pDNA-miR-223) was performed to quantify miR-223 expression in J774A.1 and peritoneal macrophages after incubation with HA-PEI/miR-223 or HA-PEI/pDNA-miR-223 NPs. Peritoneal macrophages or J774A.1 macrophages (200,000 cells per well) were plated in 6-well plates were transfected with HA-PEI/miR-223 NPs at a dosage of 100 nM miR-223 or HA-PEI/pDNA-miR-223 NPs at 20 μg DNA per 200,000 cells. miR-223 complexed with Lipofectamine^®^ iMAX was used as a control. After 6 h incubation with HA-PEI/miR-223 NPs, HA-PEI/pDNA-miR-223 NPs, blank HA-PEI NPs, Lipofectamine^®^/miR-223, the transfecting solutions were removed and replaced with fresh DMEM medium. The cells were harvested at pre-determined time points post-transfection for RNA isolation, and miR-223 expression level was quantified by Taqman^®^ miRNA assay using quantitative PCR (qPCR) (LightCycler 480, Roche, Branford, CT). U6 small RNA (snRNA) was used as a housekeeping gene.

### *In vitro* polarization studies

The ability of HA-PEI/miR-223 NPs to modulate macrophage phenotype from M1 to M2 was assessed by measuring the change in the expression level of iNOS2 (M1 marker) and Arg1 (M2 marker) in J774A.1 or peritoneal macrophages after transfection with the NPs. The J774A.1 or peritoneal macrophages (200,000 cells per well) were cultured in 6-well plates and stimulated to an M1 phenotype by overnight incubation with LPS (100 ng/mL) and IFN-*γ* (100 ng/mL). Subsequently, the M1 macrophages were transfected with HA-PEI/miR-223 or HA-PEI/pDNA-miR-223 NPs and Lipofectamine/miR-223 at a dose equivalent to 20 μg DNA or 100 nM miR-223 mimic per 200,000 cells. After 6 h of incubation, the cells were washed with PBS and cultured in DMEM medium. The cells were harvested at 48 h post-transfection with the NPs. The expression of iNOS2 and Arg1 was quantified by qPCR. β -actin was used as a house keeping gene.

### *In vitro* anti-inflammatory effect

To investigate *in vitro* anti-inflammatory effect of HA-PEI/miR-223 NPs, peritoneal macrophages (200,000 cells per well) were cultured in 6-well plates and transfected with HA-PEI/miR-223, blank HA-PEI NPs, or Lipofectamine/miR-223 at a miR-223 dose of 100 nM per 200,000 cells for 48 h, followed by stimulation with LPS (100 ng/mL) for 6 h. The cells were then harvested for RNA isolation. Gene expression of pro-inflammatory cytokines TNF-α, IL-1β, and IL-6 was quantified by qPCR. β -actin was used as a housekeeping gene.

### Data analysis

Data were expressed as mean ± standard deviation. The statistical significance of difference between experimental and control groups was determined using a student’s *t*-test. A p value of less than 0.05 was considered statistically significant.

## Results

### *In vitro* characterization of HA-PEI/miRNA nanoparticles in macrophages

**Fig 1:**
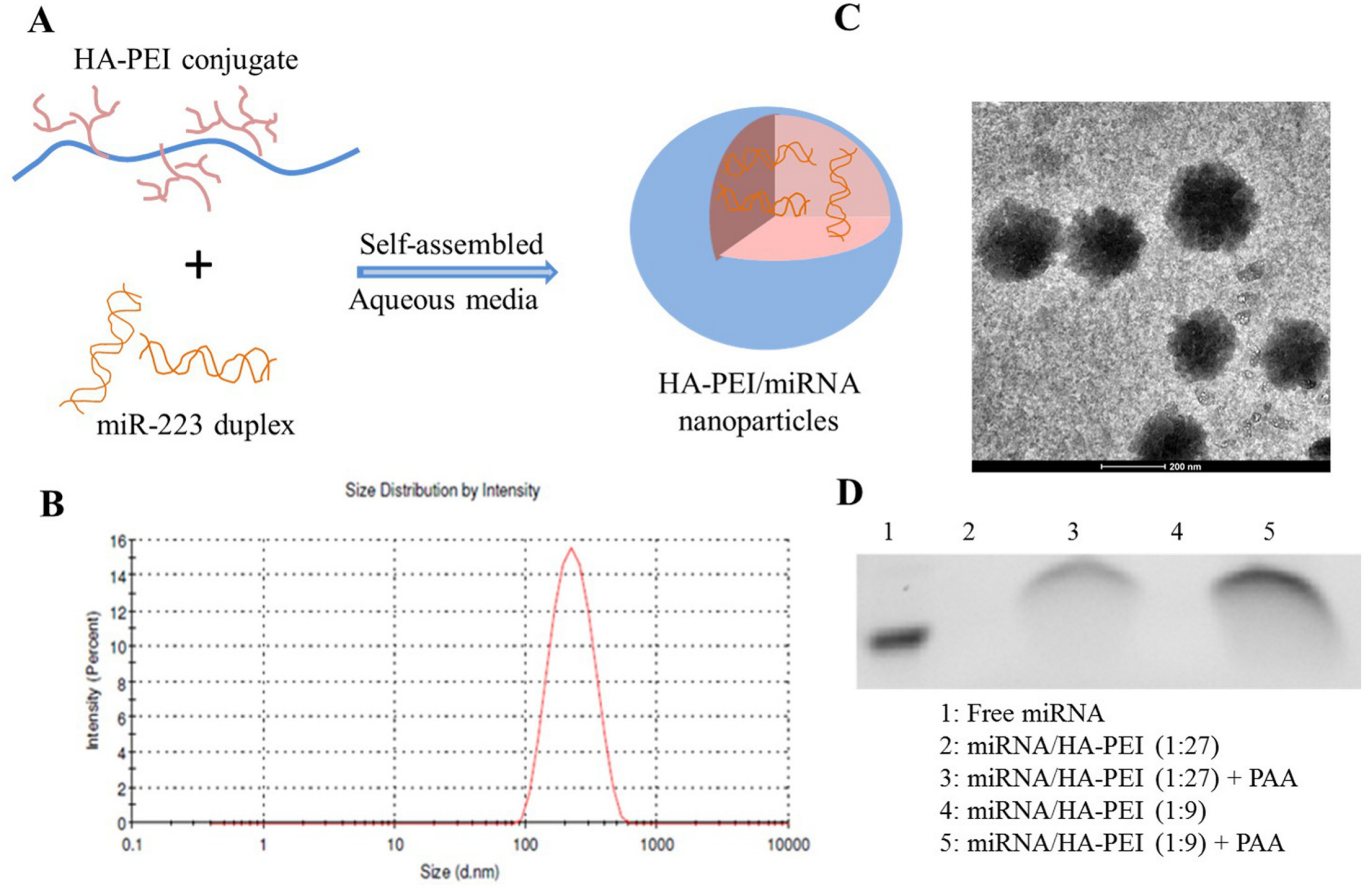
(a) Schematic illustration of complexation of microRNA-223 (miR-223) with hyaluronic acid-poly(ethyleneimine) (HA-PEI) conjugate. (b) Size distribution of HA-PEI/miR-223 (9:1) nanoparticles (NPs) in phosphate-buffered saline (pH 7.4) by dynamic light scattering. (c) Transmission electron microscopy (TEM) image of HA-PEI/miR-223 NPs in PBS (9:1). (d) Agarose electrophoretic analysis of miR-223 duplex encapsulation in HA-PEI NPs with different polymer-to-miRNA weight ratios (27:1 and 9:1 w/w).

HA-PEI conjugate was successfully conjugated for complexation with miR-223 or plasmid DNA expressing miR-223 (pDNA-miR-223) via electrostatic interaction between positively charged PEI and negatively charged nucleic acids to form nanoparticles (NPs) (Fig 1a). Particle size and morphology of HA-PEI/miR-223 NPs (9:1 ratio) were measured by DLS and TEM, respectively. The HA-PEI/miR-223 NPs showed unimodal size distribution with an average size of about 200 nm in PBS (Fig 1b). TEM showed spherical shape of HA-PEI/miR-223 NP with particle size of around 200 nm which is similar to the result obtained by DLS (Fig 1c). Both blank HA-PEI and HA-PEI/miR-223 NPs exhibited negative surface charge in PBS as reflected by the zeta potential value of -17.0 and -14.7 mV, respectively, suggesting core/shell structure of the NPs in which the core contained PEI complexed miRNA surrounded by the hydrophilic shell of negatively charged HA. The HA-PEI/miR-223 NPs were stable for at one week at room temperature without visible precipitation.

To investigate loading capacity of HA-PEI for miR-223 duplex, the miRNA in nuclease-free water was physically mixed with HA-PEI solution in PBS at different polymer-to-miRNA weight ratios (27:1, and 9:1), and run on a 4% agarose gel. Fig 1D showed the absence of miRNA band at all ratios when miRNA was encapsulated in the HA-PEI NPs (lane 2 and 4), indicating that miR-223 was completely encapsulated into HA-PEI. The ability of the complexes to release miRNA was then determined by treating the samples with competing anionic polymer such as poly(acrylic acid) (PAA) and run the samples on the agarose gel. The agarose gel showed the intact bands of miR-223 after decomplexation (lane 3 and 5). When the miRNA was completely inside the HA-PEI NPs, the band completely disappeared and it appeared after treating with PAA, indicating that miRNA was release from HA-PEI NPs in the presence with excess PAA. The result suggests that miRNA may be efficiently released from the NPs in the cells which is considered as one of the most important steps in nucleic acid delivery [19].

**Fig 2:**
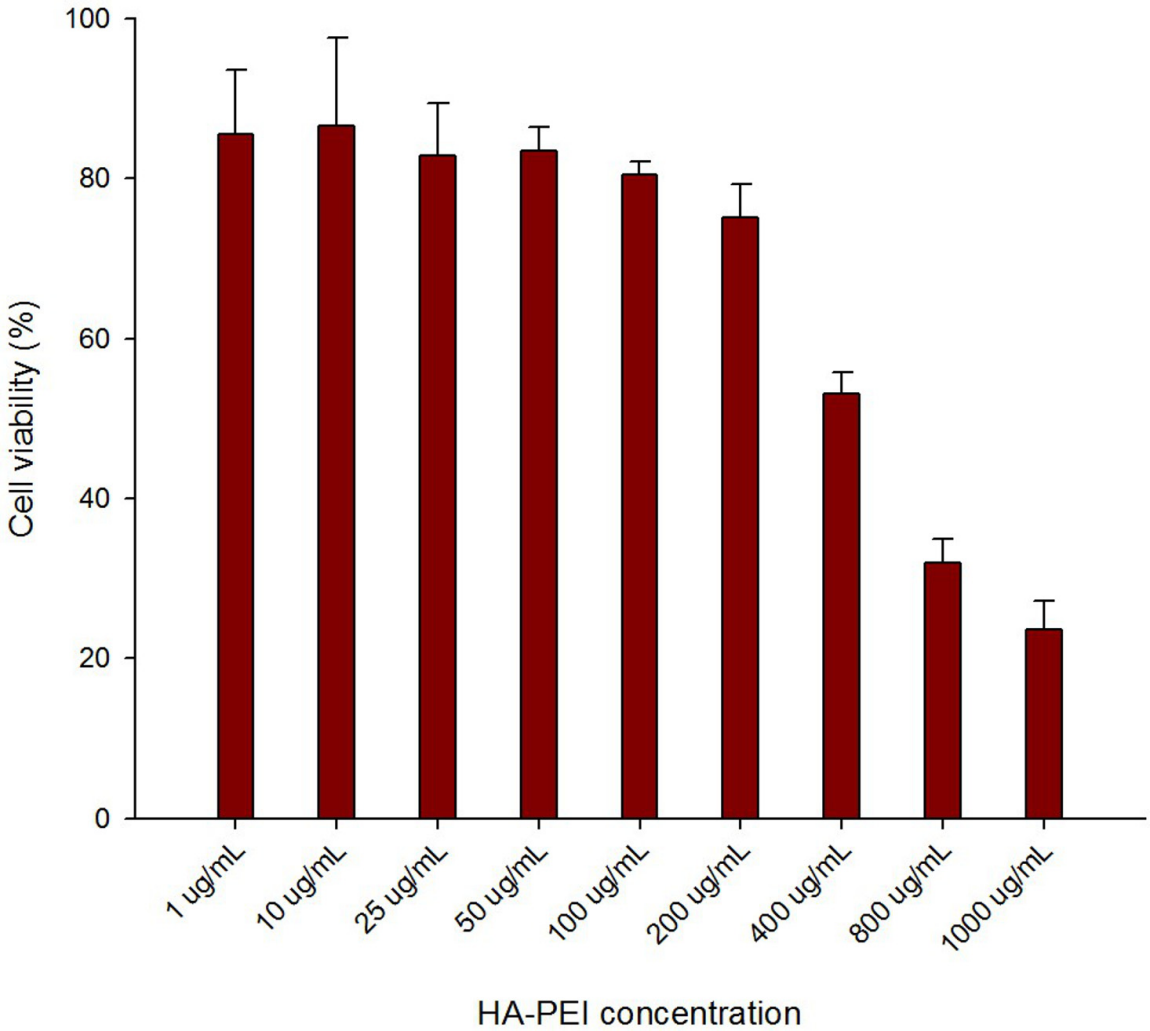
Assessment of cellular toxicity as measured by viability of J774A.1 macrophages incubated with hyaluronic acid-poly(ethyleneimine) (HA-PEI) nanoparticles at different concentrations from 1 μg/mL to 1,000 μg/mL for 24 hours.

We next evaluated cytotoxicity of HA-PEI in J774A.1 macrophages by MTT assay after 24 h incubation with different concentrations of HA-PEI ranging from 1 μg/mL to 1000 μg/mL. Fig 2 shows that cell viability was greater than 80% when concentration of HA-PEI NPs was less than 200 μg/mL, while the HA-PEI concentration used for transfection study was less than 50 μg/mL, indicating that there was no cytotoxicity of HA-PEI for macrophages during transfection studies. However, when HA-PEI concentration increased to 400 μg/mL, the cell viability decreased to about 50% and to 25% at 1000 μg/mL of HA-PEI NPs, indicating cytotoxicity of HA-PEI at high concentrations.

### Cellular uptake and miR-223 transfection in J774A.1 and peritoneal macrophages

**Fig 3:**
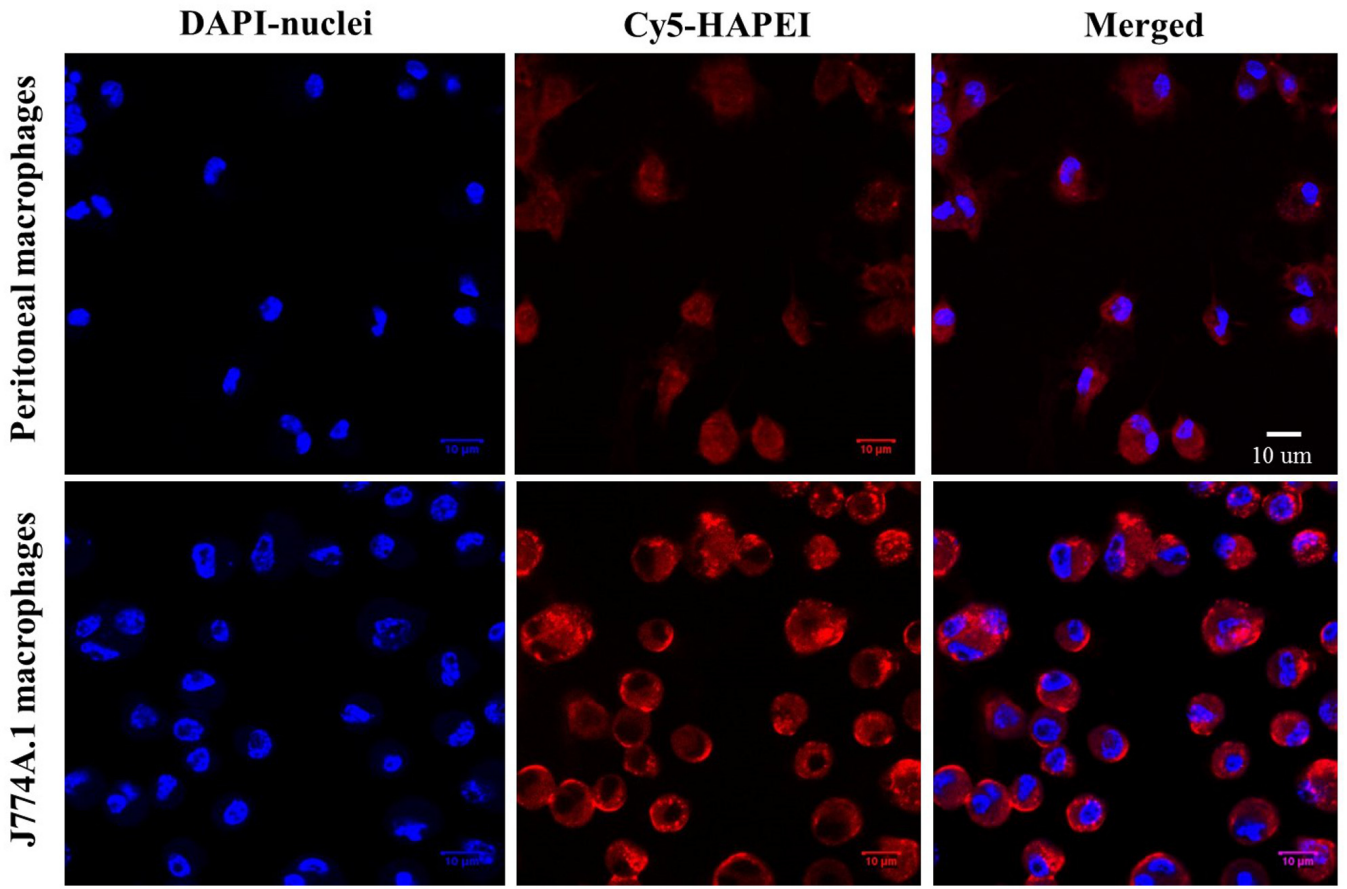
Fluorescence microscopy analysis of uptake of miR-223-encapsulated in hyaluronic acid-poly(ethyleneimine) (HA-PEI) nanoparticles in J774A.1 alveolar and primary peritoneal macrophages and macrophages at 2 h post-incubation. Prior to cellular uptake study, HA-PEI was conjugated with a red fluorescence dye, Cy5.

Cellular uptake of HA-PEI/miR-223 NPs in J774A.1 macrophages and primary peritoneal macrophages was evaluated by confocal microscopy. Bright red signal (Cy5.5) of HA-PEI was observed inside both J774A.1 and peritoneal macrophages after 2 h of incubation (Fig 3), indicating that HA-PEI/miRNA NPs were effectively internalized by both adherent murine macrophages and primary peritoneal macrophages.

**Fig 4:**
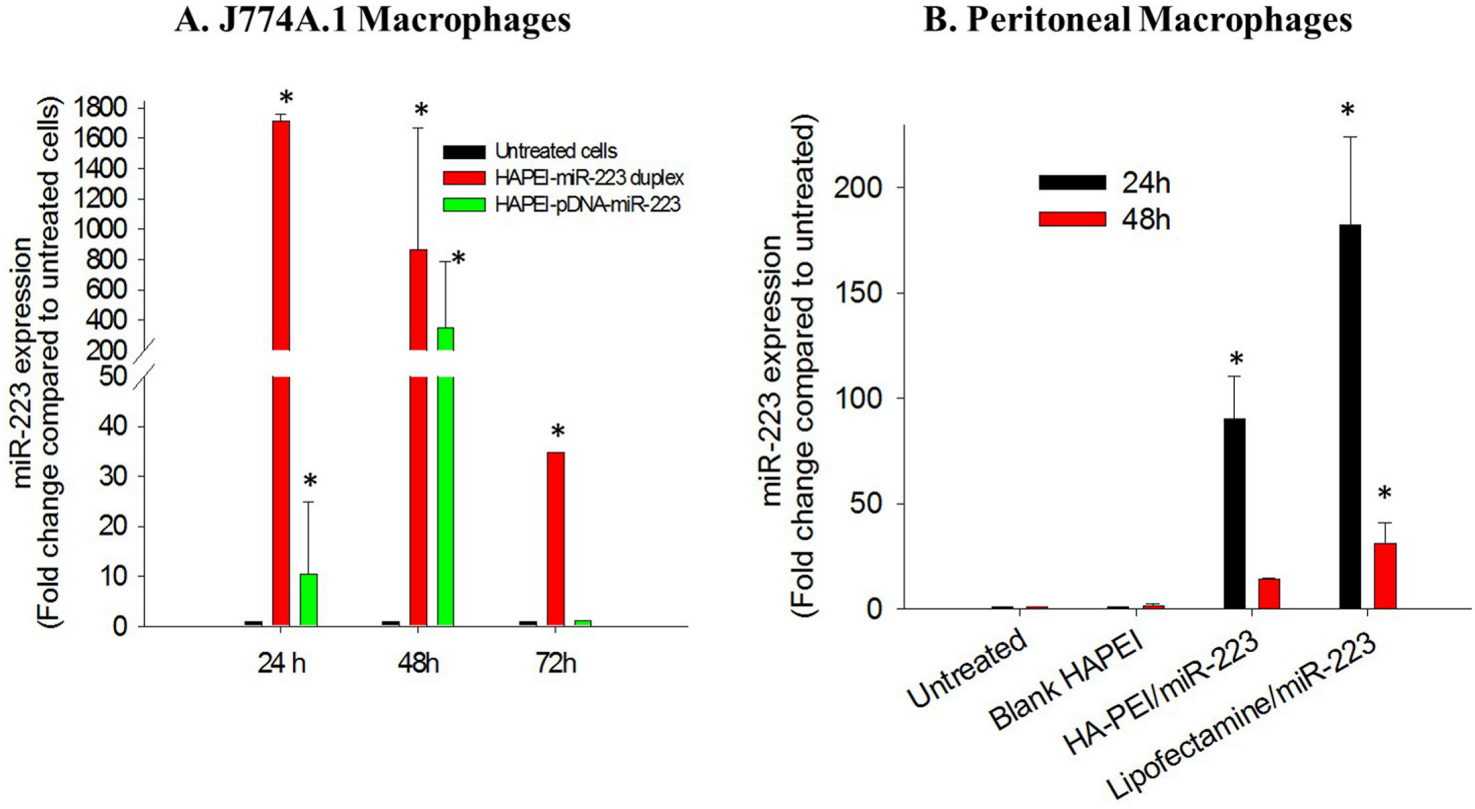
(a) Comparative transfection study of hyaluronic acid-poly(ethyleneimine) (HA-PEI) / miR-223 duplexes nanoparticles (NPs) and HA-PEI/plasmid DNA expressing miR-223 (pDNA-miR-223) NPs in J774A.1 at 24 h, 48 h, and 72 h post-transfection. (b) miR-223 expression in primary peritoneal macrophages at 24 h and 48 h post-transfection of HA-PEI/miR-223 and Lipofectamine^®^/miR-223. miR-223 expression was quantified by Taqman miRNA assay specific for miR-223; U6snRNA was used as a house keeping gene. *p<0.05 compared to untreated macrophages, n = 3.

Transfection efficiency of HA-PEI/miR-223 duplex was compared with that of HA-PEI/pDNA-miR-223 in J774A.1 macrophages. Fig 4a shows miR-223 expression in the cells upon transfection with HA-PEI/miR-223 duplex or PEI/pDNA-miR-223 NPs for 24 h, 48h, and 72 h. miR-223 mediated transfection dramatically increased miR-223 expression in the cells from 24 h and lasted to 72 h with highest expression level of 1,600-fold compared to the control at 24 h post-transfection. miR-223 expression level decreased to 800-fold at 48 h and to 35-fold at 72 h post-miR-223 transfection. On the contrary, miR-223 expression induced by pDNA-miR-223 mediated transfection reached highest level of only 200-fold at 48 h post-transfection and decreased to basal level at 72 h. Increasing the pDNA-miR-223 dose did not result in an increase in miR-233 expression in the cells (data not shown). The results indicated that transfection efficiency of miR-223 was much better and lasted longer than that of pDNA-miR-223 in J774A.1 macrophages using the same delivery vehicle.

The ability of HA-PEI NPs to efficiently deliver miR-223 duplex was further evaluated in peritoneal macrophages. Fig 4b shows miR-223 gene expression levels in peritoneal macrophages after transfection with HA-PEI/miR-223 and Lipofectamine^®^/miR-223 at 24 h and 48 h post-transfection. HA-PEI and Lipofectamine^®^ (a commercialized cationic lipid-based transfection agent) mediated transfection increased miR-223 expression to 100 fold and 170 fold at 24 h post-transfection, respectively, indicating efficient transfection of miR-223 in peritoneal macrophages by HA-PEI nanoparticles. The results also showed that highest level of miR-223 expression in peritoneal macrophages upon transfection with HA-PEI/miR-223 duplex was much lower than that in J774A.1, indicating lower transfection efficiency of HA-PEI/miR-223 in primary macrophages than that in adherent murine macrophages. Furthermore, it should be noted that Lipofectamine^®^ was very toxic to macrophages; a significant number of cells died after 6 h of incubation with Lipofectamine^®^/miR-223 while the cells transfected with HA-PEI/miR-223 were healthy throughout the study (as indicated in the MTT assay), indicating that HA-PEI was a suitable carrier for miRNA delivery to macrophages.

### *In vitro* repolarization study

**Fig 5:**
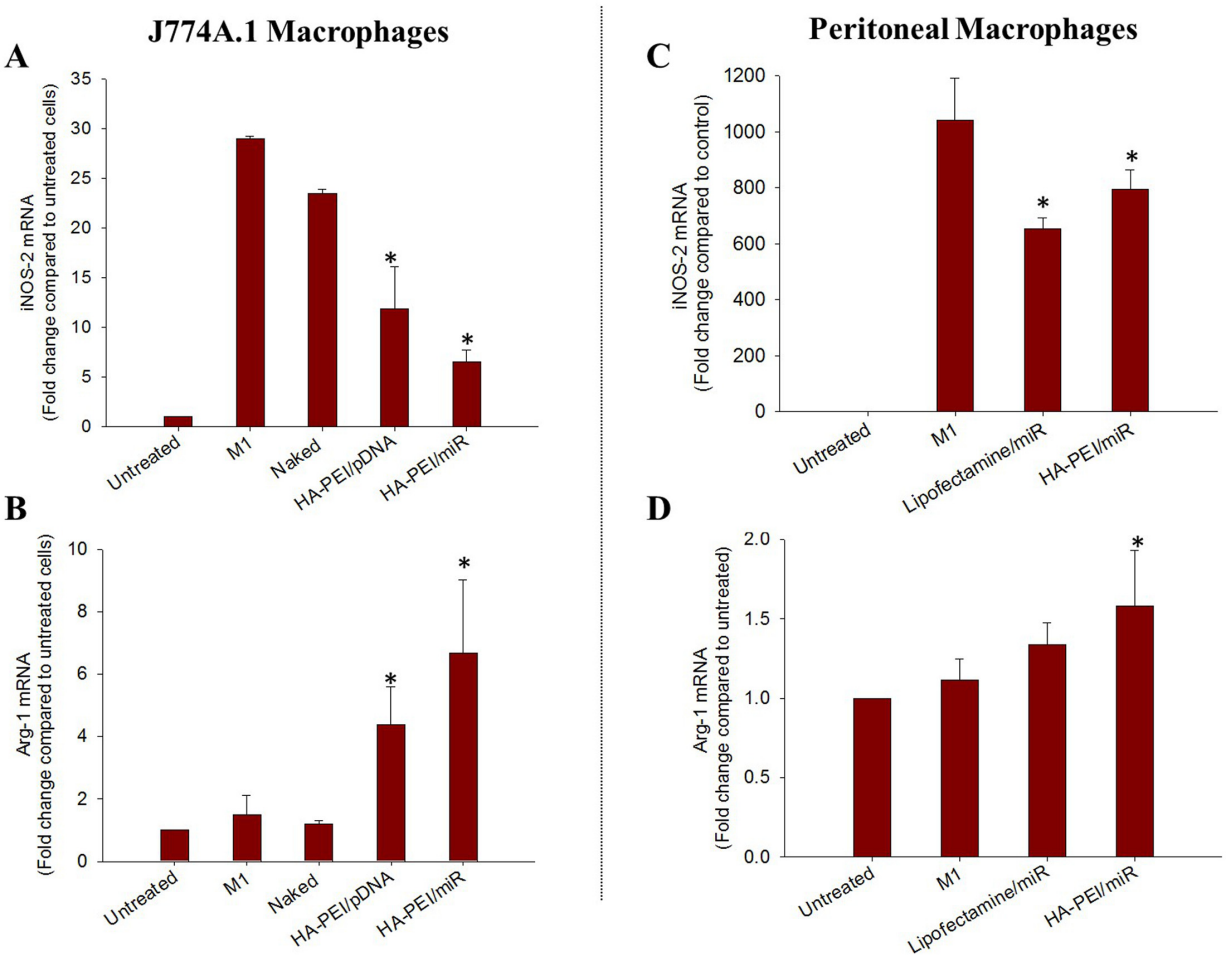
*In vitro* polarization study. (a) iNOS-2 (M1 marker) and Arg-1 (M2 marker) in M1 J774A.1 macrophages treated with hyaluronic acid-poly(ethyleneimine) (HA-PEI)/miR-223 duplexes nanoparticles (NPs) or HA-PEI/plasmid DNA expressing miR-223 ((pDNA-miR-223) NPs for 48 h. (b) iNOS-2 (M1 marker) and Arg-1 (M2 marker) in M1 peritoneal macrophages treated with HA-PEI/miR-223 or Lipofectamine^®^/miR-223 for 48 h. *p<0.05 compared to M1 macrophages which were obtained by stimulating macrophages with lipopolysaccharide (LPS, 100 ng/mL) combined with interferon-gamma (100 ng/mL) for 16 h, n = 3.

In the previous study, a simple system to define M1 and M2 macrophages was established in which iNOS-2 was defined as M1 marker and Arg-1 was defined as M2 marker [15]. To investigate if high expression of miR-223 in macrophages could re-polarize macrophages from M1 to M2 phenotypes, qPCR was used to measure iNOS and Arg gene expression level in the M1 macrophages transfected with HA-PEI/miR-223 for 48 h in J774A.1 and peritoneal macrophages (Fig 5). In J774A.1 macrophages, LPS and IFN-γ stimulation increased iNOS level to 30-fold compared to untreated cells, indicating M1 phenotype. Transfection of M1 cells with HA-PEI/miR-223 and HA-PEI/pDNA-miR-223 NPs significantly decreased iNOS level to 12 fold and 6 fold compared to untreated cells, respectively (Fig 5a). Furthermore, treatment of the M1 cells with HA-PEI/miR-223 and HA-PEI/pDNA-miR-223 NPs increased Arg level to 6.5 and 4.5 fold compared to untreated and M1 cells, respectively (Fig 5b). The results indicated that J774A.1 macrophages were effectively re-polarized from M1 to M2 state by miR-223 duplex and pDNA-miR-223 encapsulated in HA-PEI NPs. In addition, HA-PEI/miR-223 showed lower iNOS and higher Arg level in the M1 cells than HA-PEI/pDNA-miR-223 at 48 h post-treatment, indicating better polarizing effect by miR-223 duplex than pDNA-miR-223, which was due to higher miR-223 expression in the cells by miR-223 mediated transfection than that by pDNA-miR-223. Macrophage polarizing effect of miR-223 duplex was then evaluated in primary peritoneal macrophages. The cells were first stimulated to M1 phenotype with LPS and IFN-γ for 16 h, followed by transfection with miR-223 duplex complexed with HA-PEI or Lipofectamine^®^. M1 peritoneal macrophages had high iNOS expression level of 1,000-fold but no change in Arg expression compared to untreated cells. Treatment of M1 peritoneal macrophages with HA-PEI/miR-223 and Lipofectamine^®^/miR-223 significantly decreased iNOS level to 750-fold and 600-fold compared to untreated cells, respectively. In addition, HA-PEI/miR-223 increased Arg expression to 1.5-fold as compared to the untreated cells. The results indicated that miR-223 formulations shifted peritoneal macrophages spectrum from M1 to M2 phenotype (Fig 5c and 5d).

### *In vitro* anti-inflammatory effect of HA-PEI/miR-223 NPs in peritoneal macrophages

**Fig 6:**
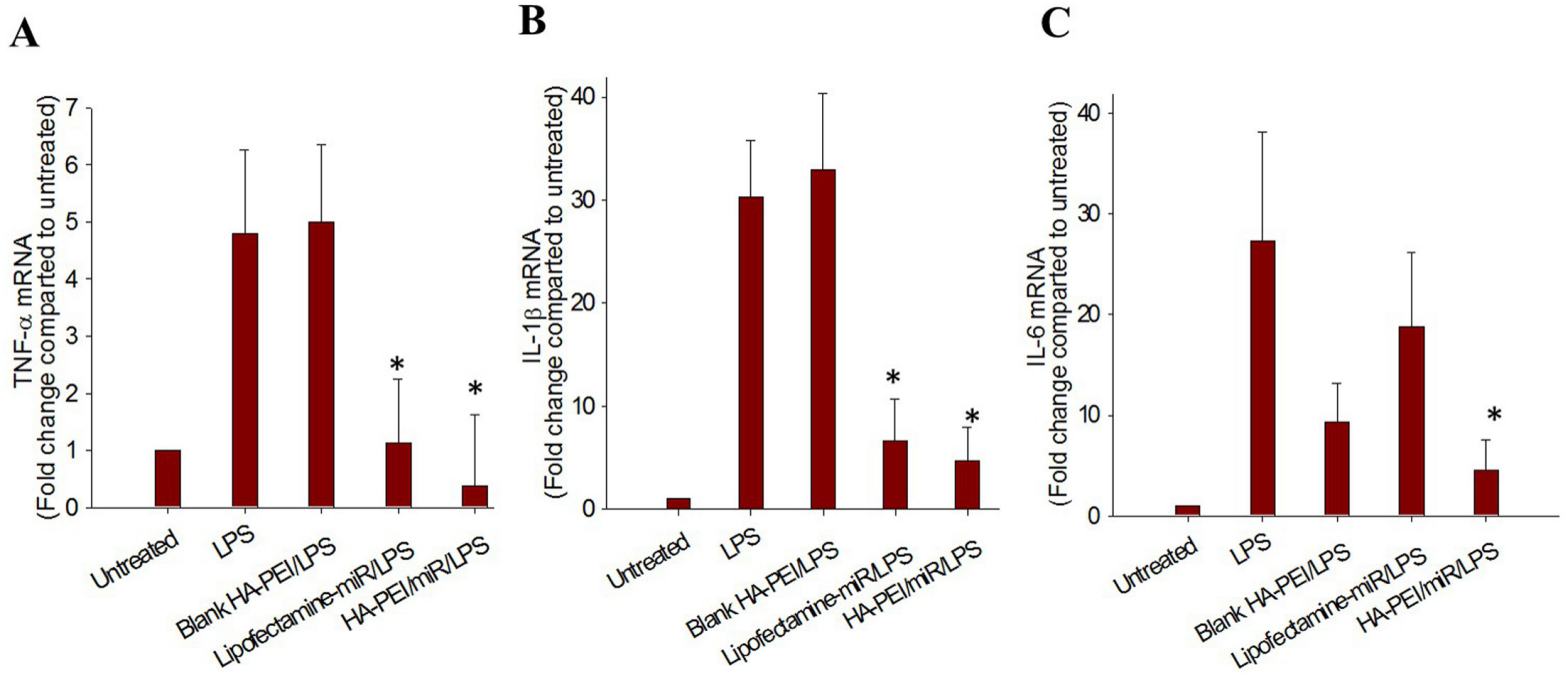
*In vitro* anti-inflammatory study in peritoneal macrophages: expression of pro-inflammatory cytokines (a) TNF-α, (b) IL-1β, and (c) IL-6 mRNA level at 48 h post-transfection of macrophages with hyaluronic acid-poly(ethyleneimine) (HA-PEI)/miR-223 duplexes nanoparticles (NPs) or Lipofectamine^®^/miR-223, followed by stimulation with LPS (100 ng/mL) for 6 h. qPCR was used to quantify mRNA levels. *p<0.05 compared to LPS-treated group, n = 3.

Following the switch of macrophages to the M2 phenotype, we investigated *in vitro* anti-inflammatory effect of HA-PEI/miR-223 NPs in peritoneal macrophages. The macrophages were transfected with HA-PEI/miR-223 NPs for 48 h before stimulation with LPS for 6 h. The LPS stimulation upregulated TNF-α, IL-1β, and IL-6 in un-transfected peritoneal macrophages to 5 fold, 30 fold, and 27 fold, respectively, compared to control cells (Fig 6a–6c). Treatment with blank HA-PEI NPs did not change the level of TNF-α and IL-1β, but decreased IL-6 level for currently unknown reason compared to LPS-stimulated cells without treatment. However, when peritoneal macrophages were pre-transfected with HA-PEI/miR-223 NPs or Lipofectamine^®^/miR-223, TNF-α, IL-1β and IL-6 gene expression levels was significantly decreased with more pronounced effect by HA-PEI/miR-223 NPs. For example, treatment with HA-PEI/miR-223 NPs decreased TNF-α, IL-1β and IL-6 gene expression levels to 0.5, 4, and 3.9 fold compared to the control, respectively, indicating that HA-PEI/miR-223 NPs effectively suppressed the inflammation caused by LPS in peritoneal macrophages.

## Discussion

Systemic delivery of miRNA as well as other nucleic acids to macrophages is very challenging due to their highly degradative phagocytic, endosomal, and lysosomal compartments and unstable properties of nucleic acids [20–22]. In this study, HA-PEI conjugate was designed for delivery of miR-223 duplex to macrophages due to the high capacity of PEI for encapsulation of nucleic acids and targeting ability of HA to CD44-overexpressed cancer cells and macrophages [13,23,24]. As shown in Fig 1, the conjugate had high capacity for miR-223 encapsulation due to the abundant amine groups of PEI to condense miR-223 by electrostatic interactions. However, the toxicity of this cationic polymer has limited its utility for *in vivo* gene therapy applications. Therefore, conjugation of PEI with HA was expected to reduce PEI’s cytotoxicity. HA-PEI conjugate showed minimal cytotoxicity in macrophages at HA-PEI concentration less than 200 μg/mL. Our previous study showed high expression of CD44 on the membrane of J774A.1 macrophages and peritoneal macrophages [15]. The high expression of CD44 on the macrophage membrane confirmed the potential target of these cells for HA-based nucleic acid delivery. *In vitro* cellular uptake study showed effective internalization of HA-PEI/miR-223 NPs by both J774A.1 and primary peritoneal macrophages, possibly via CD44 receptor recognition which might promote the interaction of the cell membrane with the NPs, leading to enhanced endocytosis [25]. Once HA-PEI/miR-223 NPs were inside macrophages, endosome escape and miR-223 release may be facilitated by well-known proton-sponge effect of PEI to achieve successful transfection [26]. Transfection studies showed that HA-PEI/miR-223 NPs produced high and prolonged expression of miR-223 in J774A.1 macrophages, indicating that HA-PEI was an effective delivery system for targeting miRNA to macrophages. Furthermore, comparison of transfection efficiency of miRNA with that of plasmid DNA expressing the miRNA has not been previously reported. In our study, transfection of J774A.1 macrophages with HA-PEI/miR-223 induced significantly higher miR-223 expression in the cells than that with HA-PEI/pDNA-miR-223, indicating that miR-223 duplex was much better than pDNA-miR-223 for overexpression of miR-223 in macrophages.

Macrophages can switch their polarity to M1 or M2 phenotype with characteristic expression profiles of surface markers as well as cytokines and chemokines depending on the presence of stimuli in the local environment [27]. In the previous study, we showed that J774A.1 macrophages stimulated with LPS and IFN-γ polarized to M1 phenotype with high expression of iNOS, TNF-α, and CD80. In contrast, IL-4 stimulation of J774A.1 macrophages induced M2 macrophages with high expression of Arg, IL-10, CD206 and CD163 receptors [15]. In current study, we found that transfection of stimulated J774A.1 macrophages with HA-PEI/miR-223 and HA-PEI/pDNA-miR-223 NPs could re-polarize the macrophages from M1 to M2 phenotype by decreasing iNOS and increasing Arg expression. Furthermore, due to higher expression of miR-223 by miR-223 duplex mediated transfection, the macrophage polarizing effect of HA-PEI/miR-223 was better than that of HA-PEI/pDNA-miR-223. This effect of miR-223 was further confirmed in primary peritoneal macrophages. The results indicated successful repolarization of macrophages toward an anti-inflammatory phenotype by miR-223. The macrophage polarizing effect by miR-223 might be due to down-regulation of Pknox1 in macrophages, which suppressed pro-inflammatory cytokine IL-1β expression by LPS while augmenting Arg1 expression in macrophages [9]. It should be noted that transfection efficiency and polarizing effect of HA-PEI/miR-223 NPs in established J774A.1 macrophages were higher than those in primary peritoneal macrophages, implying the difficulty of macrophage targeting *in vivo*.

Following the macrophage phenotype switch, we investigated *in vitro* anti-inflammatory effect of HA-PEI/miR-223 in peritoneal macrophages. We found that pre-treatment with HA-PEI/miR-223 NPs or Lipofectamine^®^/miR-223 significantly reduced gene expression of pro-inflammatory cytokines such as TNF-α, IL-1β and IL-6 levels, indicating that the inflammation caused by LPS was effectively suppressed by HA-PEI/miR-223 NPs. The complete knockdown of of miR-223 in mice has been shown to increase inflammatory response in adipose tissues with increased macrophage infiltration and high expression of pro-inflammatory cytokines in these tissues [9]. In the current study, overexpression of miR-223 by HA-PEI NPs could induce macrophage phenotypic change toward the anti-inflammatory phenotype and subsequently reducing inflammation, suggesting potential of miR-223 therapy for the treatment of inflammatory diseases.

## Conclusions

In this study, we successfully demonstrated the macrophage re-polarization from pro-inflammatory M1 to anti-inflammatory phenotype by miR-223 encapsulated in hyaluronic acid-based nanoparticles (HA-PEI). miR-223 encapsulated HA-PEI NPs were efficiently internalized by both J774A.1 macrophages and primary peritoneal macrophages. Importantly, *in vitro* transfection of macrophages with HA-PEI/miR-223 NPs produced higher miR-223 expression level in the cells than that with HA-PEI/pDNA-miR-223 NPs, which could decrease iNOS-2 and increase Arg-1 expression level in LPS and IFN-γ stimulated J774A.1 and peritoneal macrophages. Following macrophage phenotype switch, transfection with HA-PEI/miR-223 NPs reduced inflammation in peritoneal macrophages caused by LPS by decreasing expression level of pro-inflammatory cytokines TNF-α, IL-1β, and IL-6. The results indicated successful delivery of miR-223 to macrophages and modulation of macrophage phenotype by HA-PEI NPs for anti-inflammatory effect.

## Supporting Information

S1 Data (PDF)
