# Supplementary material for: MicroRNA-223 Induced Repolarization of Peritoneal Macrophages Using CD44 Targeting Hyaluronic Acid Nanoparticles for Anti-Inflammatory Effects
Source: PLoS One. 2016 May 5;11(5):e0152024. doi: 10.1371/journal.pone.0152024 (PMC4858219; doi:10.1371/journal.pone.0152024)

Potential of microRNA-223 for polarization of macrophages to M2 phenotype

Characterization of miR-223/HAPEI NPs

			Size (d.nm):	% Intensity:	St Dev (d.n...
Z-Average (d.nm):	200.6	Peak 1:	235.0	100.0	82.06
PdI:	0.266	Peak 2:	0.000	0.0	0.000
Intercept:	0.931	Peak 3:	0.000	0.0	0.000
Result quality :	Good				

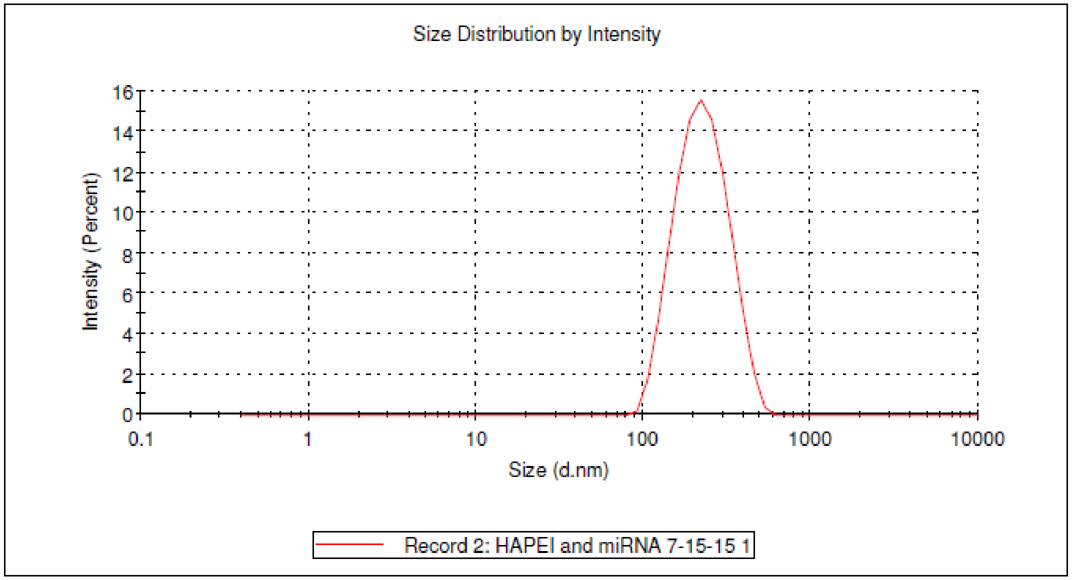

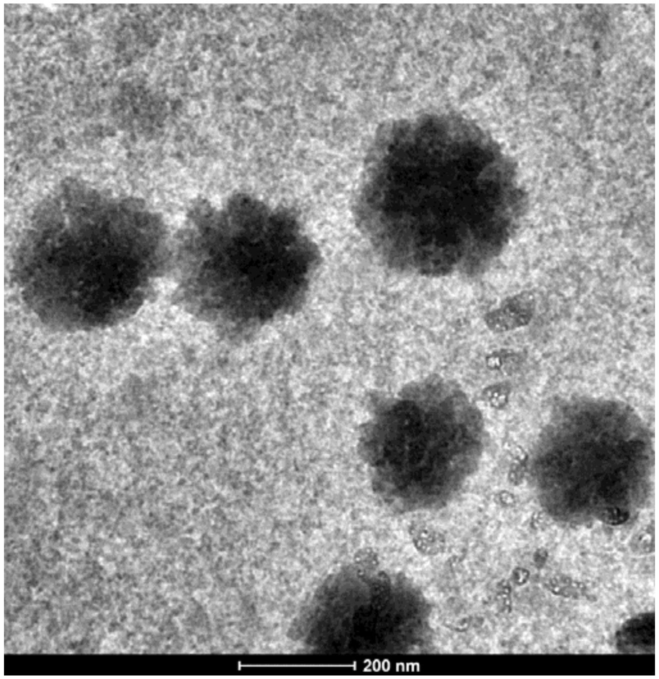

Expression level of miR-223 in M1 (LPS+IFN-γ treated) and M2 (IL4 treated) macrophages (J774A.1)

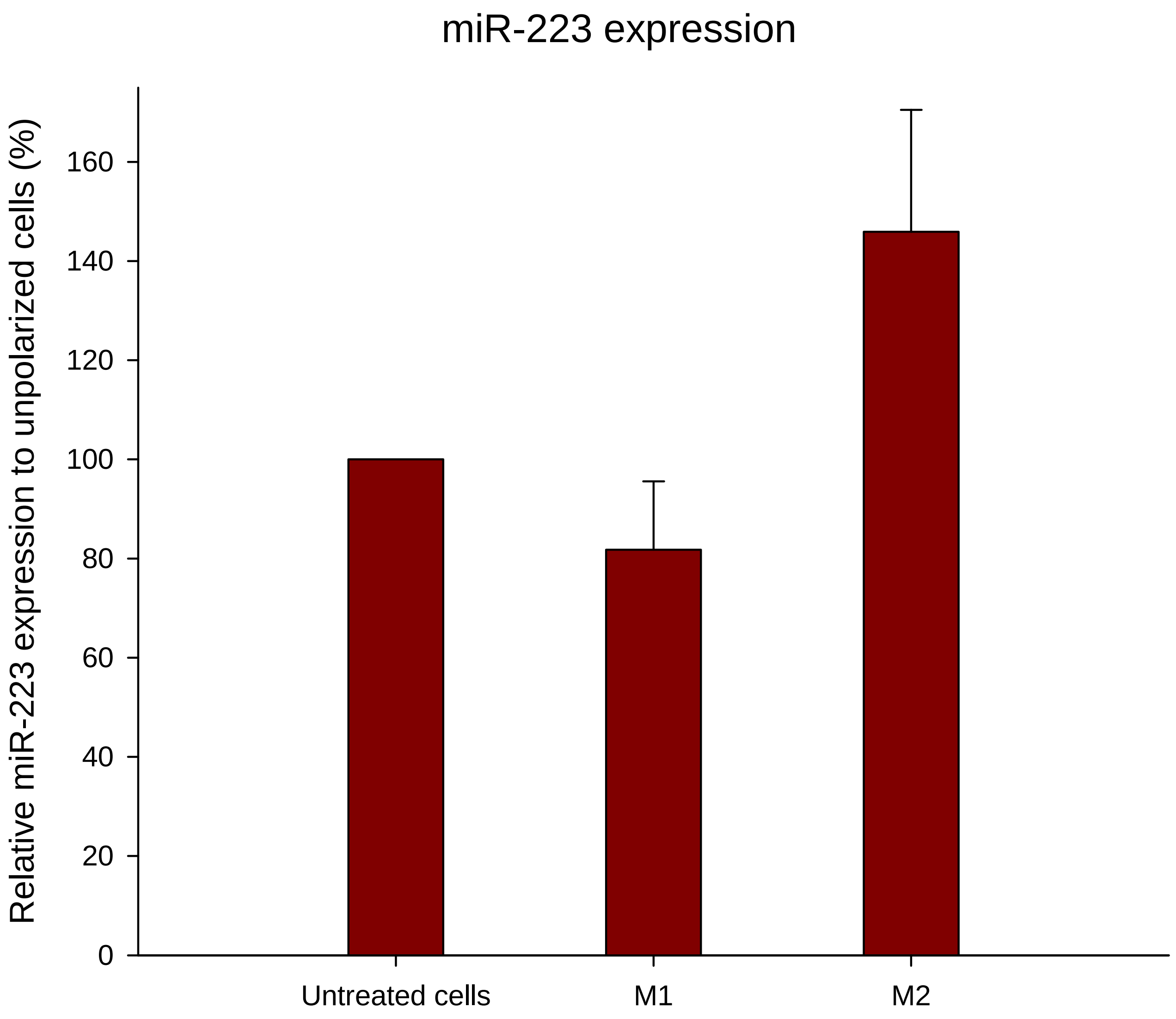

Polarization of M1 macrophages using miR-223-lipofectamin for 24 h in J774A.1

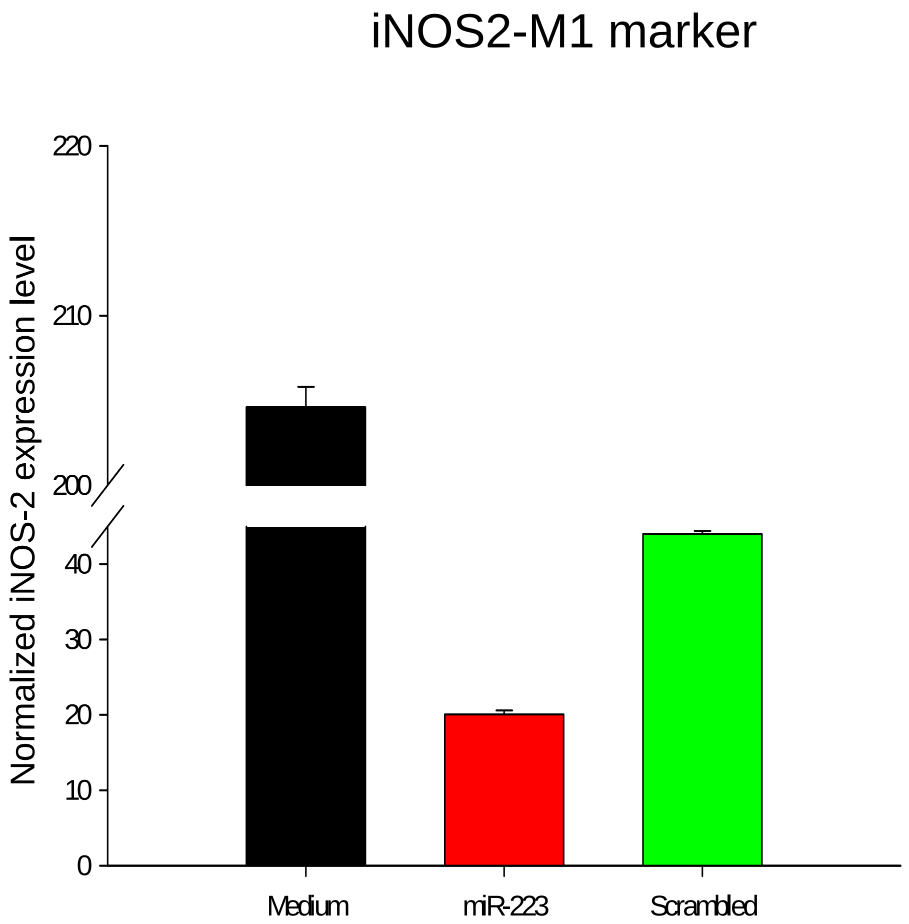

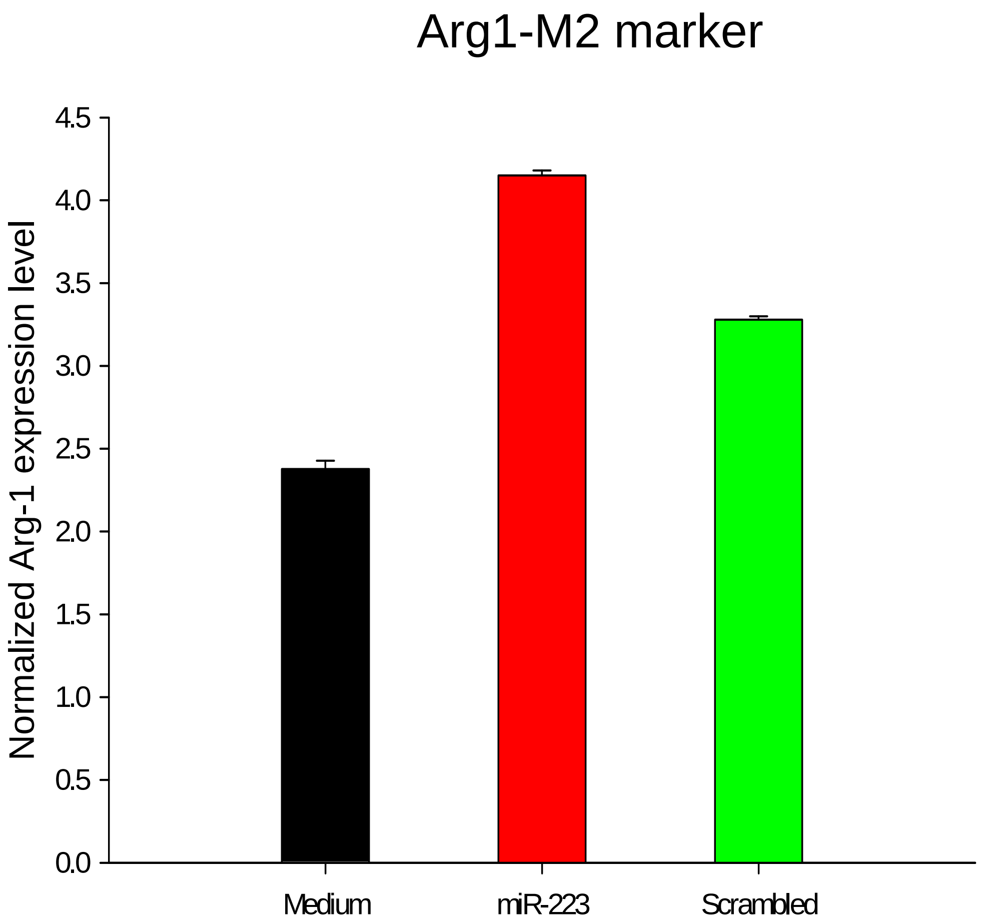

Transfection of miR-223 and plasmid-miR223 in J774A.1 macrophages-Taqman assay of miR-223

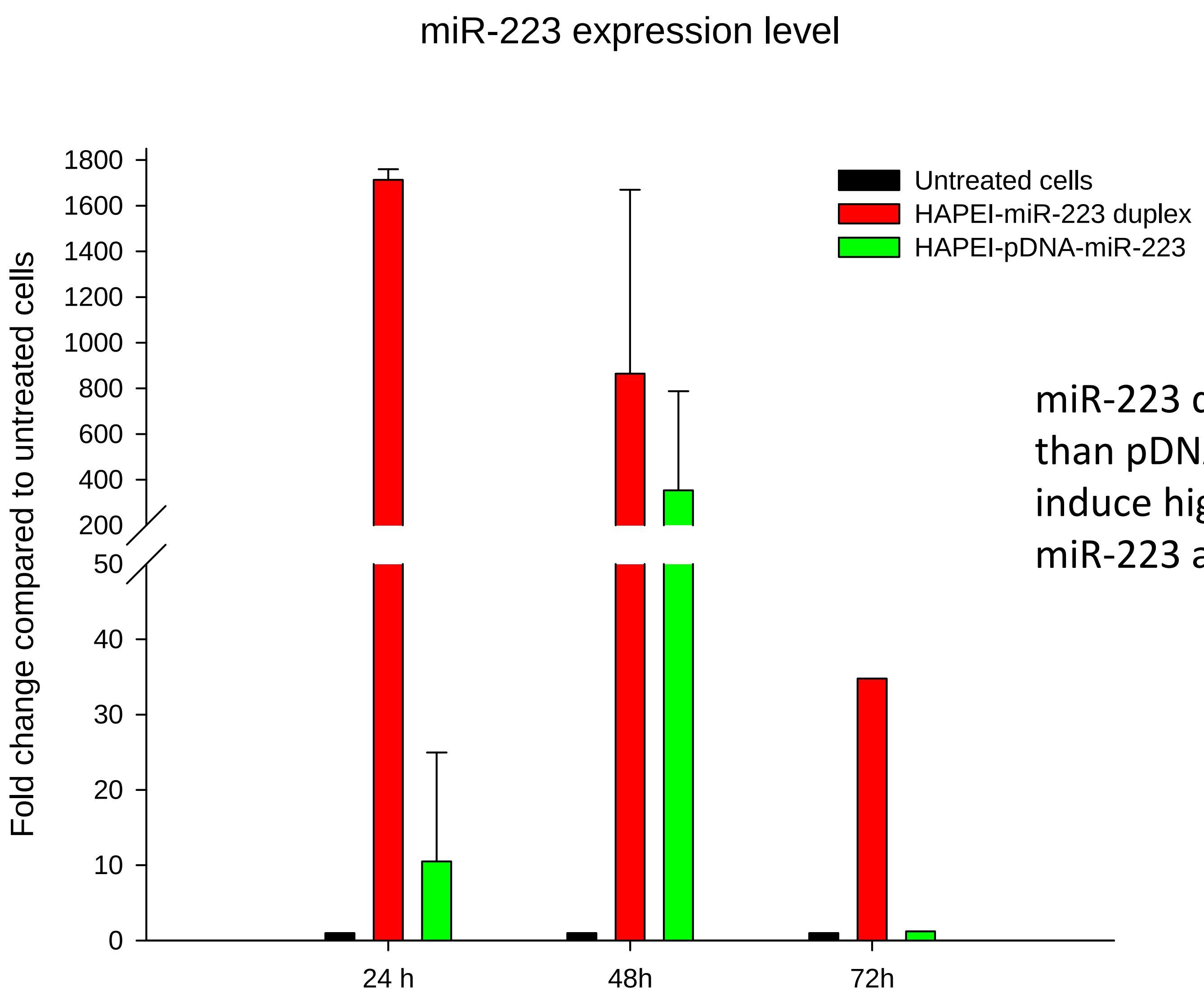

miR-223 duplex was better than pDNA-miR-223 to induce high expression of miR-223 after transfection

Transfection study of miR-223 duplex at different doses in J774A.1

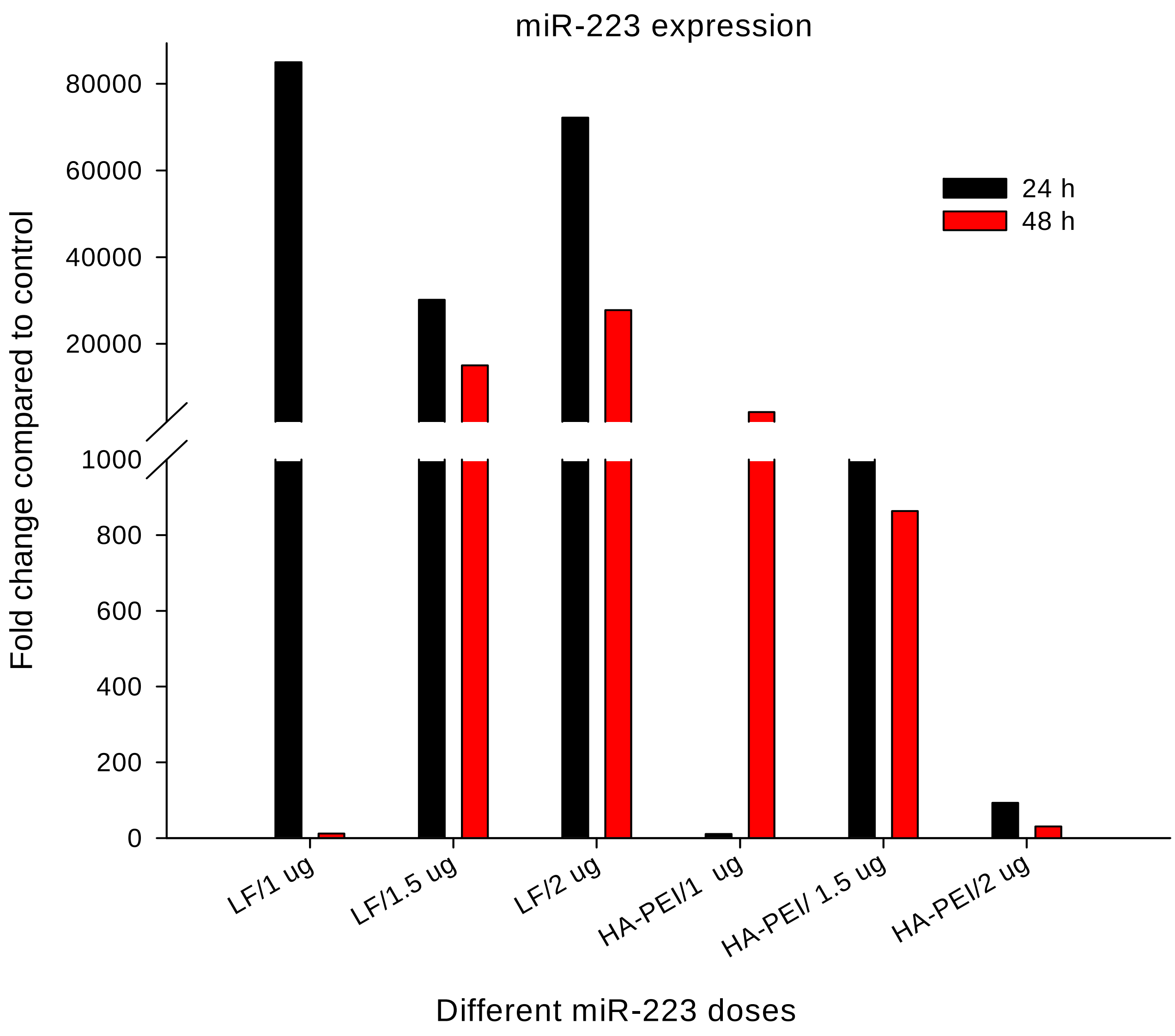

- •No correlation of transfected dose and miR-223 expression
- •Transfection efficiency of lipofectamine was better than that of HA-PEI
- •Transfection with miR-223 induced much higher miRNA expression than with pDNA-miRNA

Transfection study of pDNA-miR-223 duplex at different doses in J774A.1 macrophages

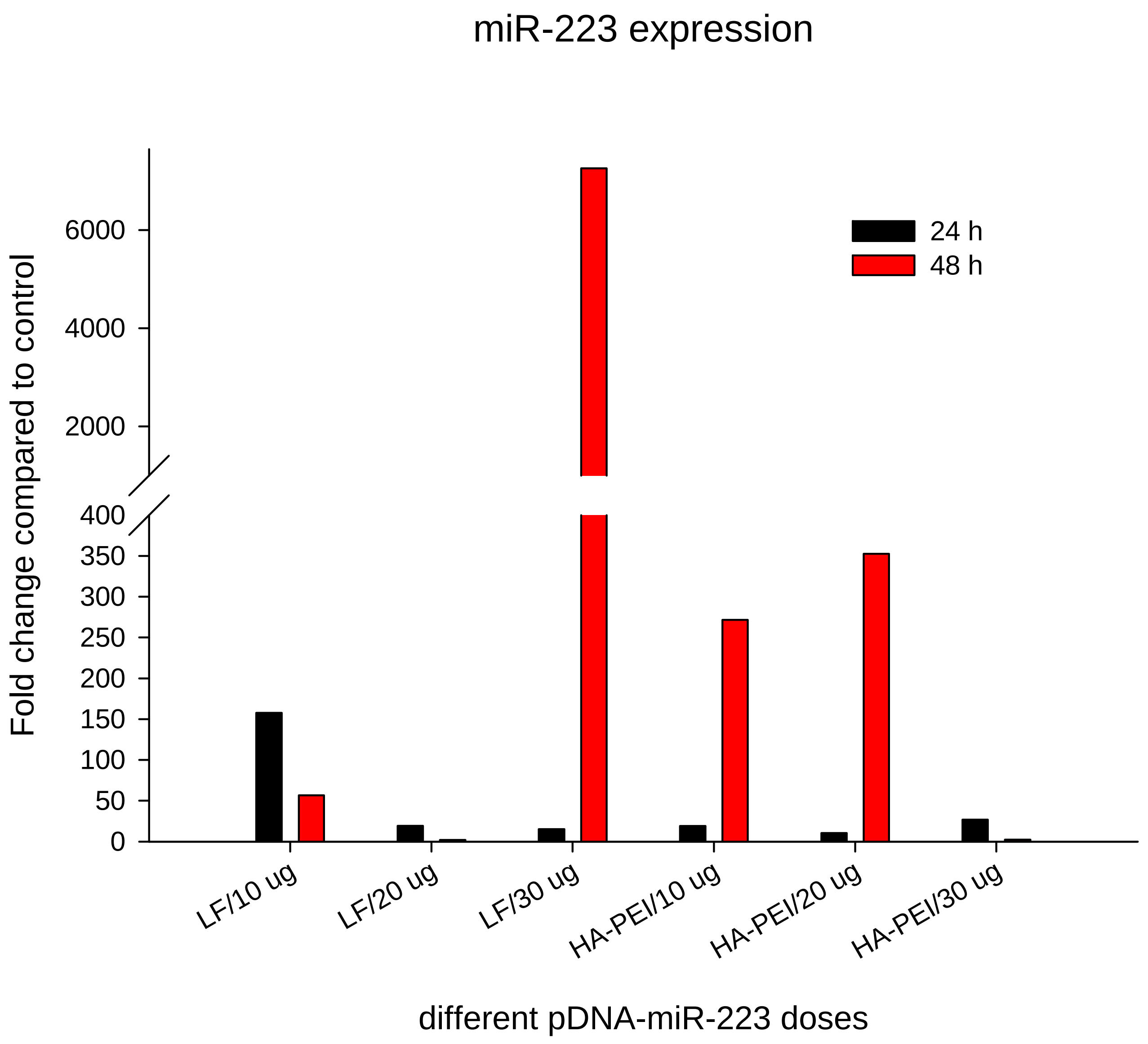

In vitro polarization of miR-223 and plasmid-miR223 (24h) in J774A.1 macrophages

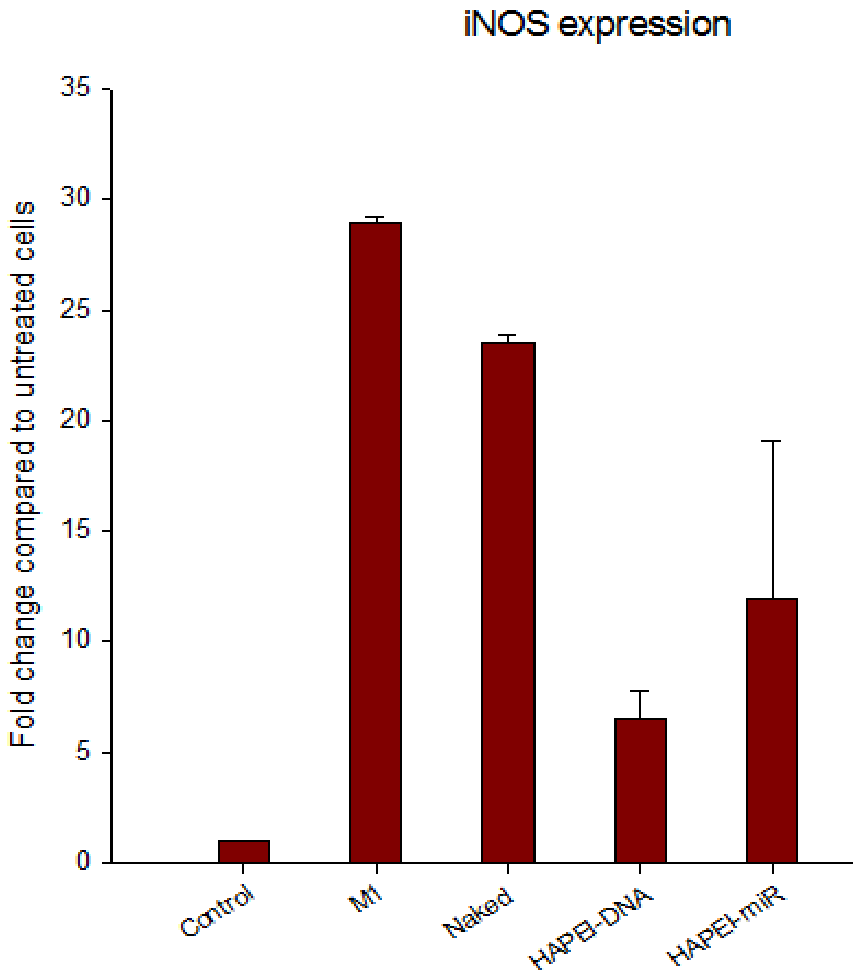

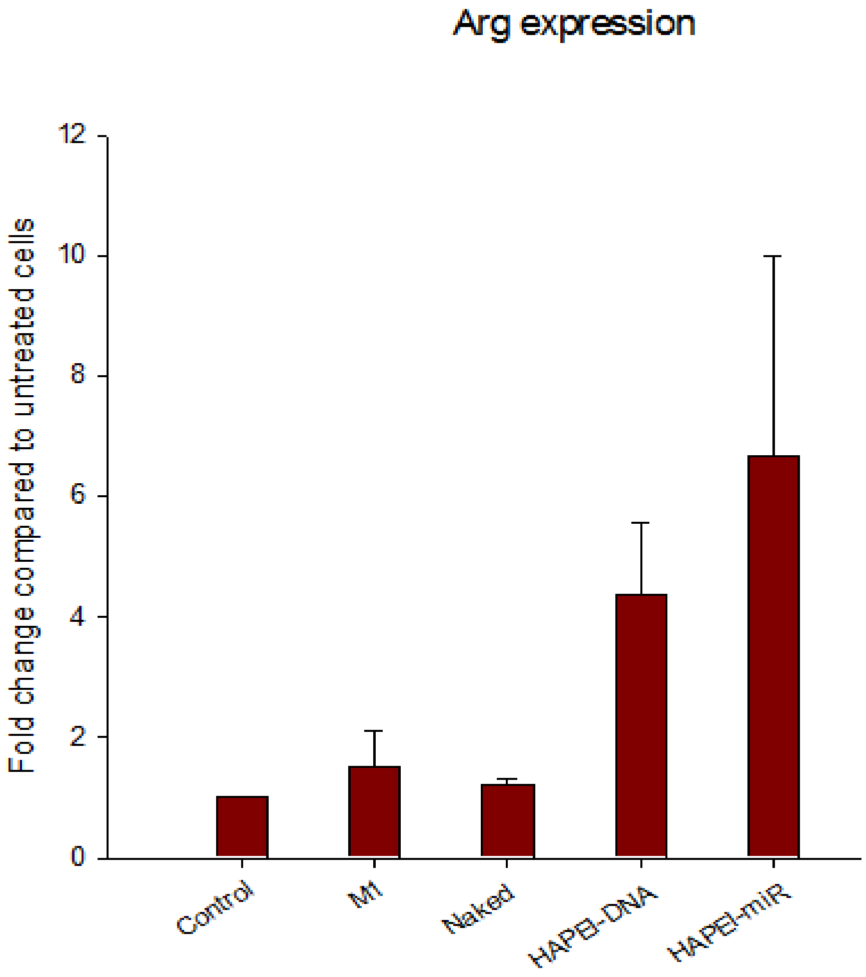

Polarization of J774 with HA-PEI/plasmid-miR-223

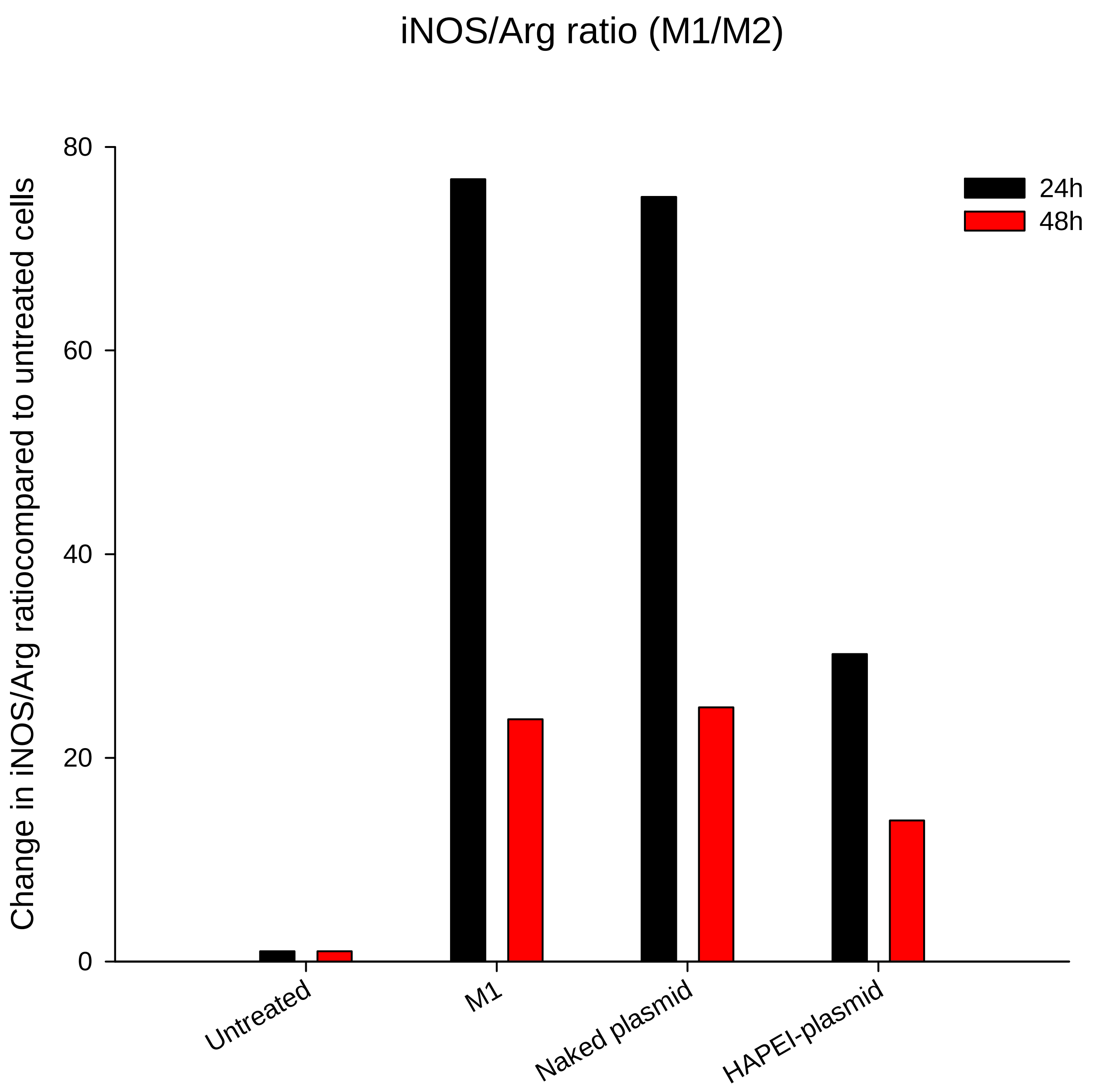

Plasmid miR-223 significantly decreased level of iNOS, other than increase Arginase level

Anti-inflammatory effect of pDNA miR-223 in J774 cells

Pre-transfected J774 with pDNA miR-223 for 24h, then treated with LPS and IFN-gamma for 6h

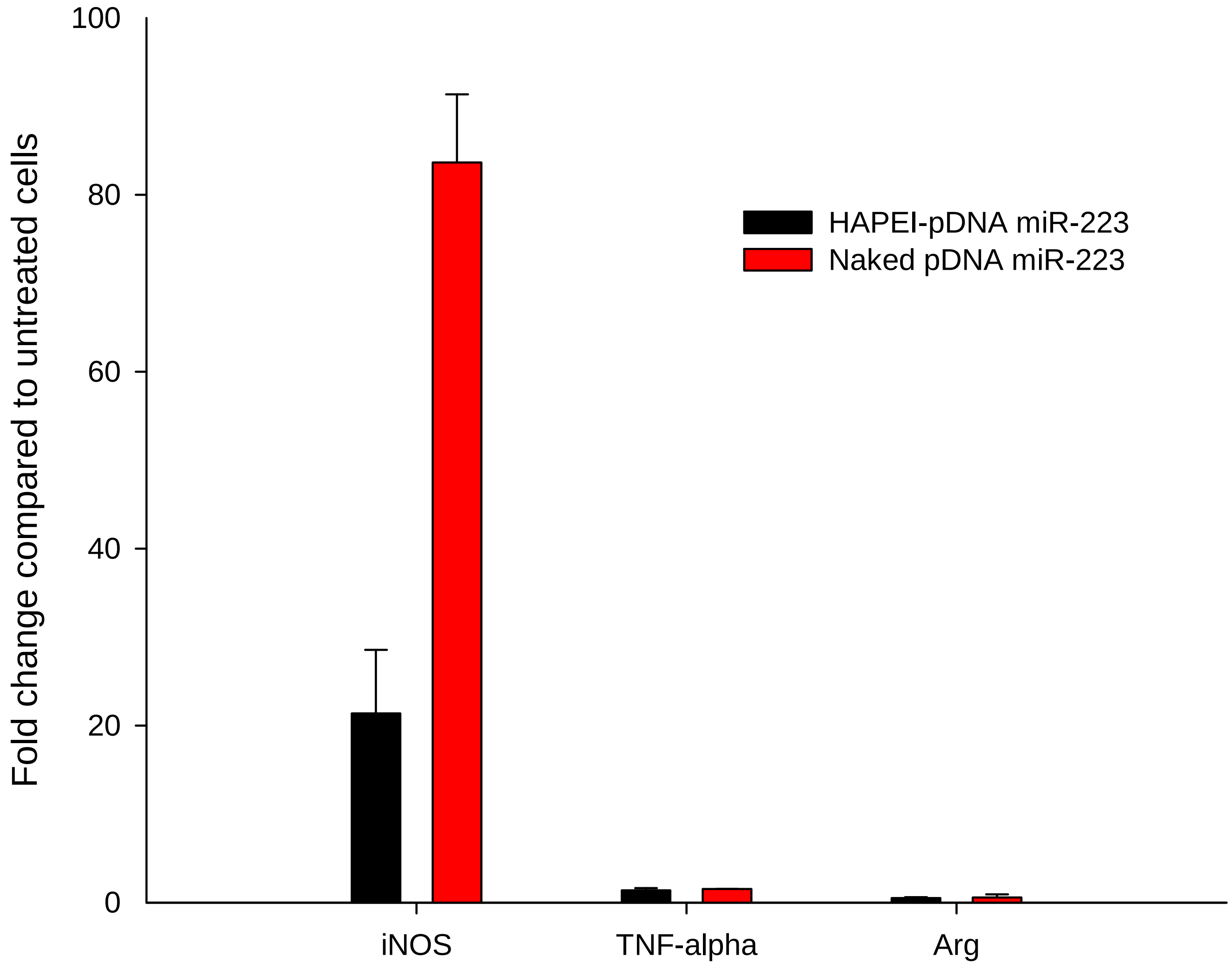

Studies in peritoneal macrophages- a better model of macrophages

Uptake of Cy5-HAPEI/miR-223 in peritoneal macrophages

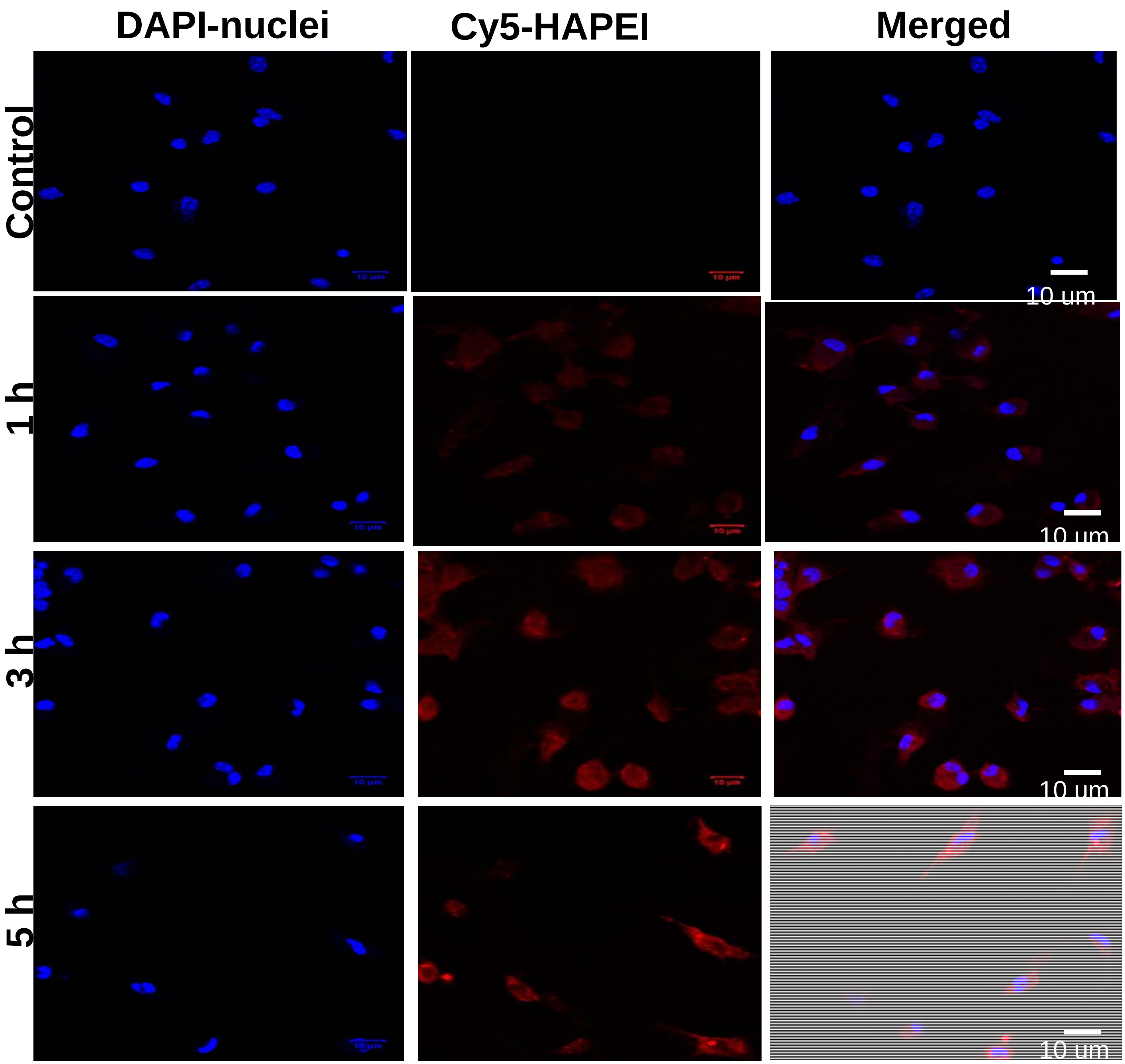

Transfection study in peritoneal macrophages

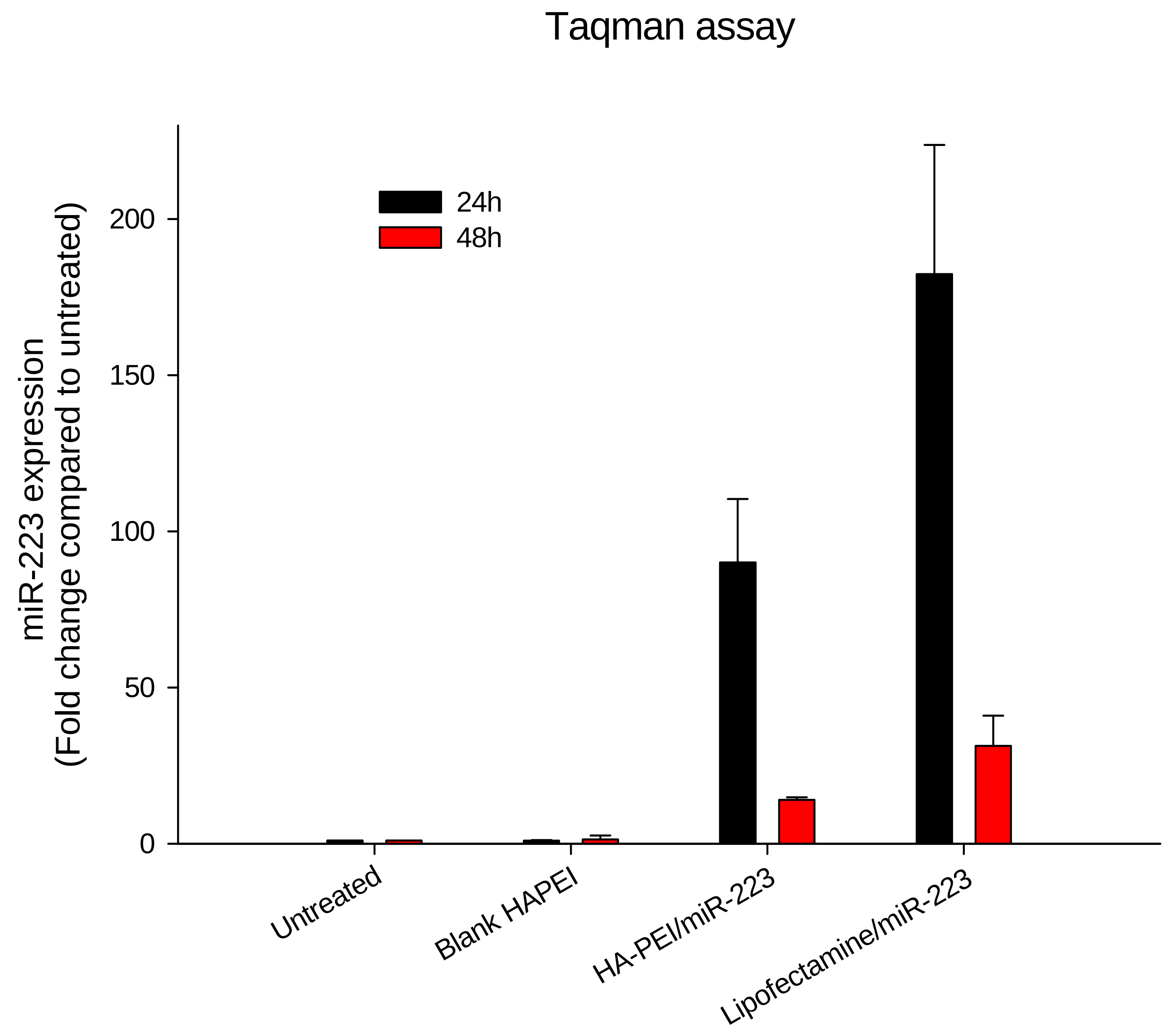

Polarization study of miR-223 (100 nM) in peritoneal macrophages

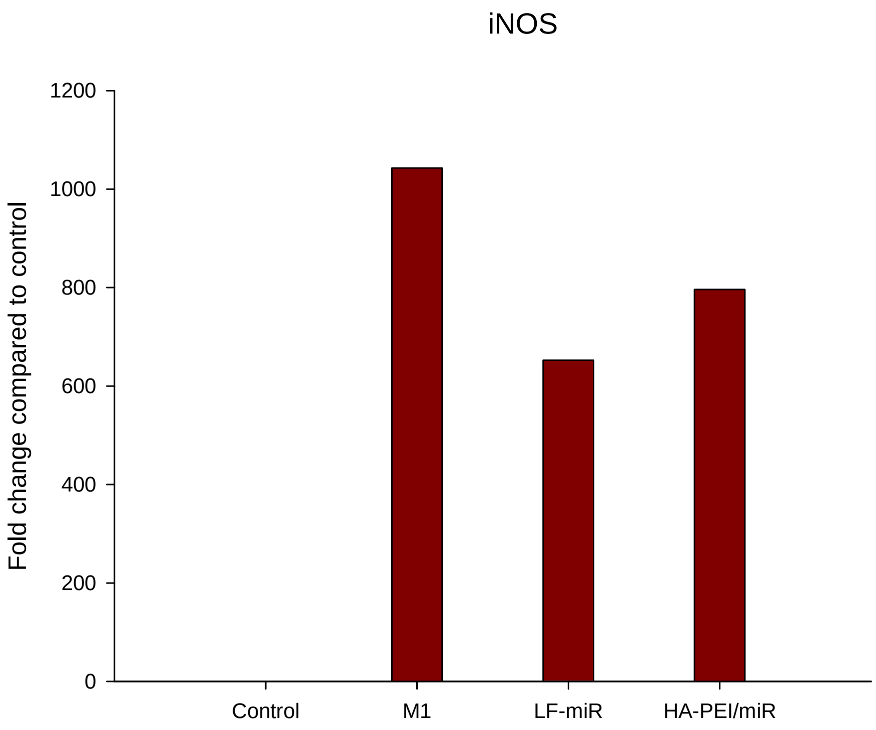

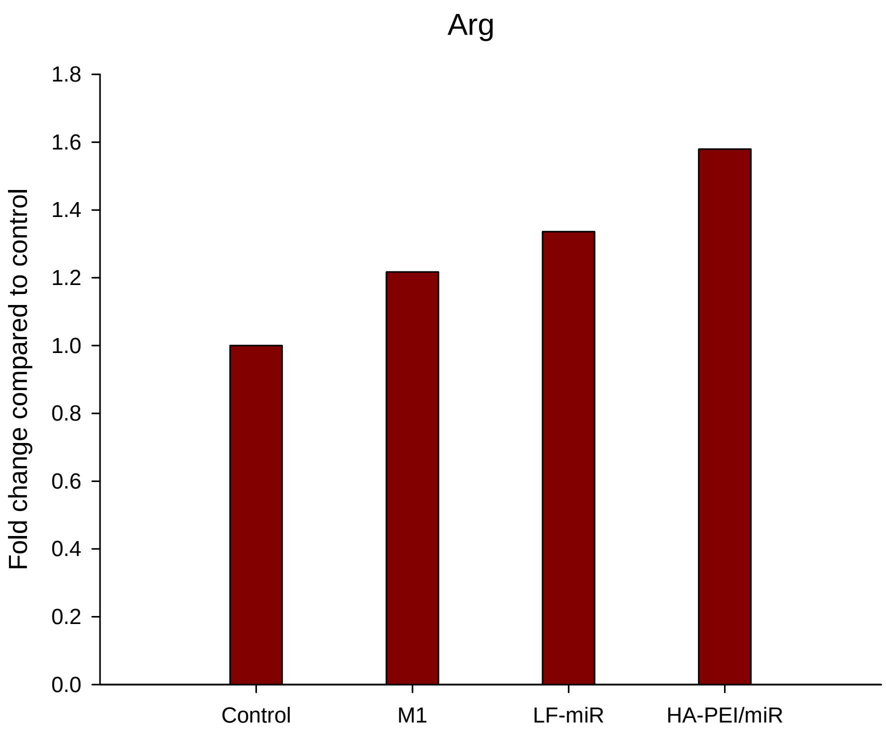

In vitro anti-inflammatory effect in peritoneal macrophages at 48h post-transfection

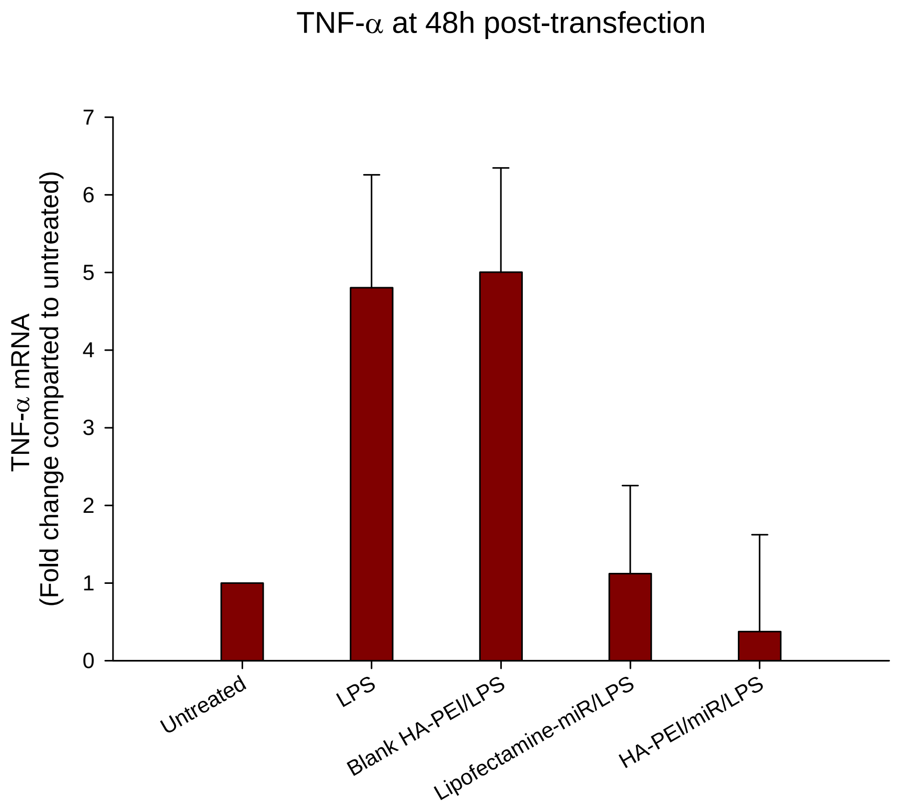

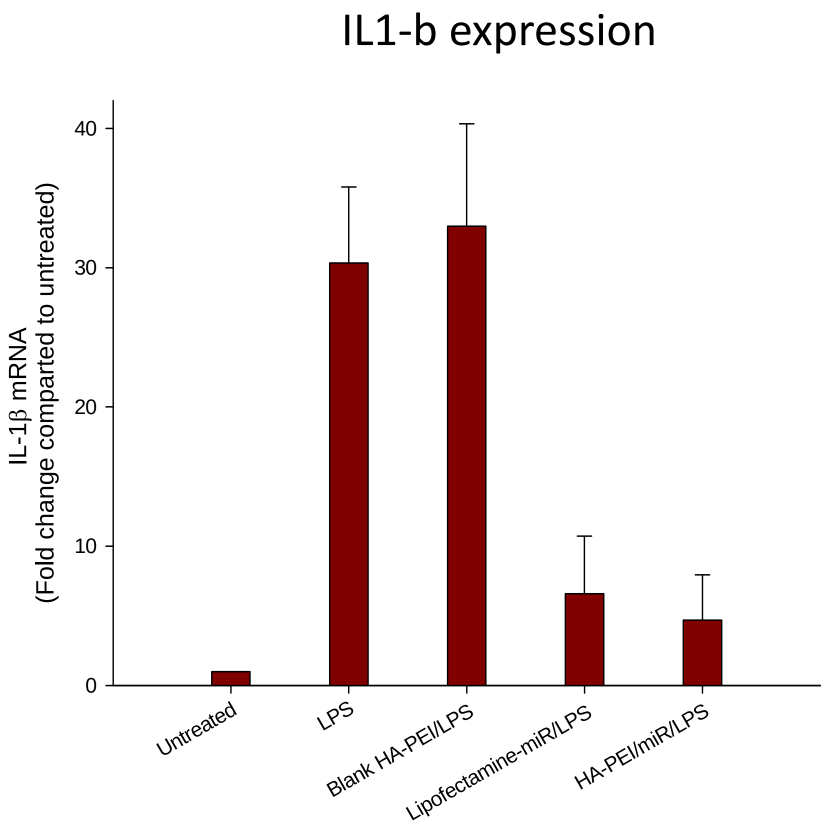

In vitro anti-inflammatory effect in peritoneal macrophages at 48h post-transfection

IL6 expression

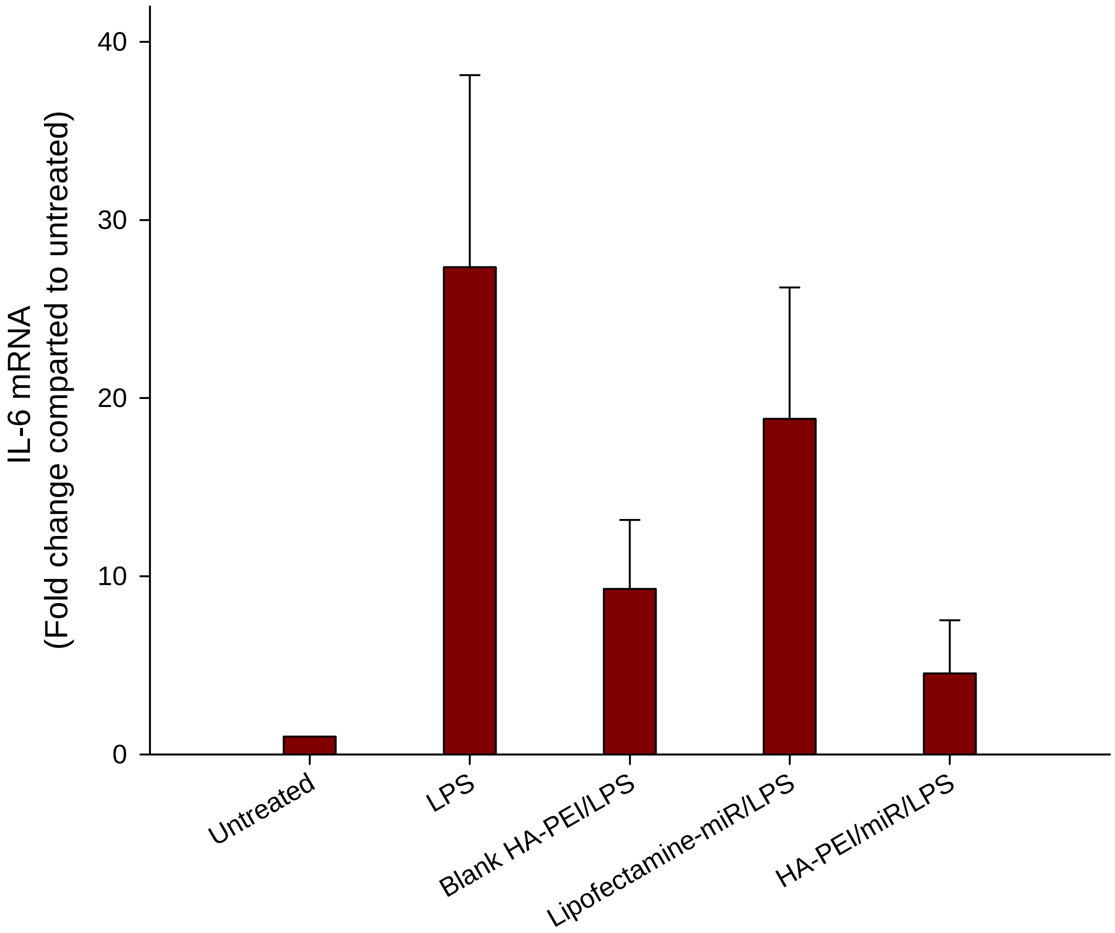

In vivo polarization study in C57BL/6 mice

peritoneal macrophages

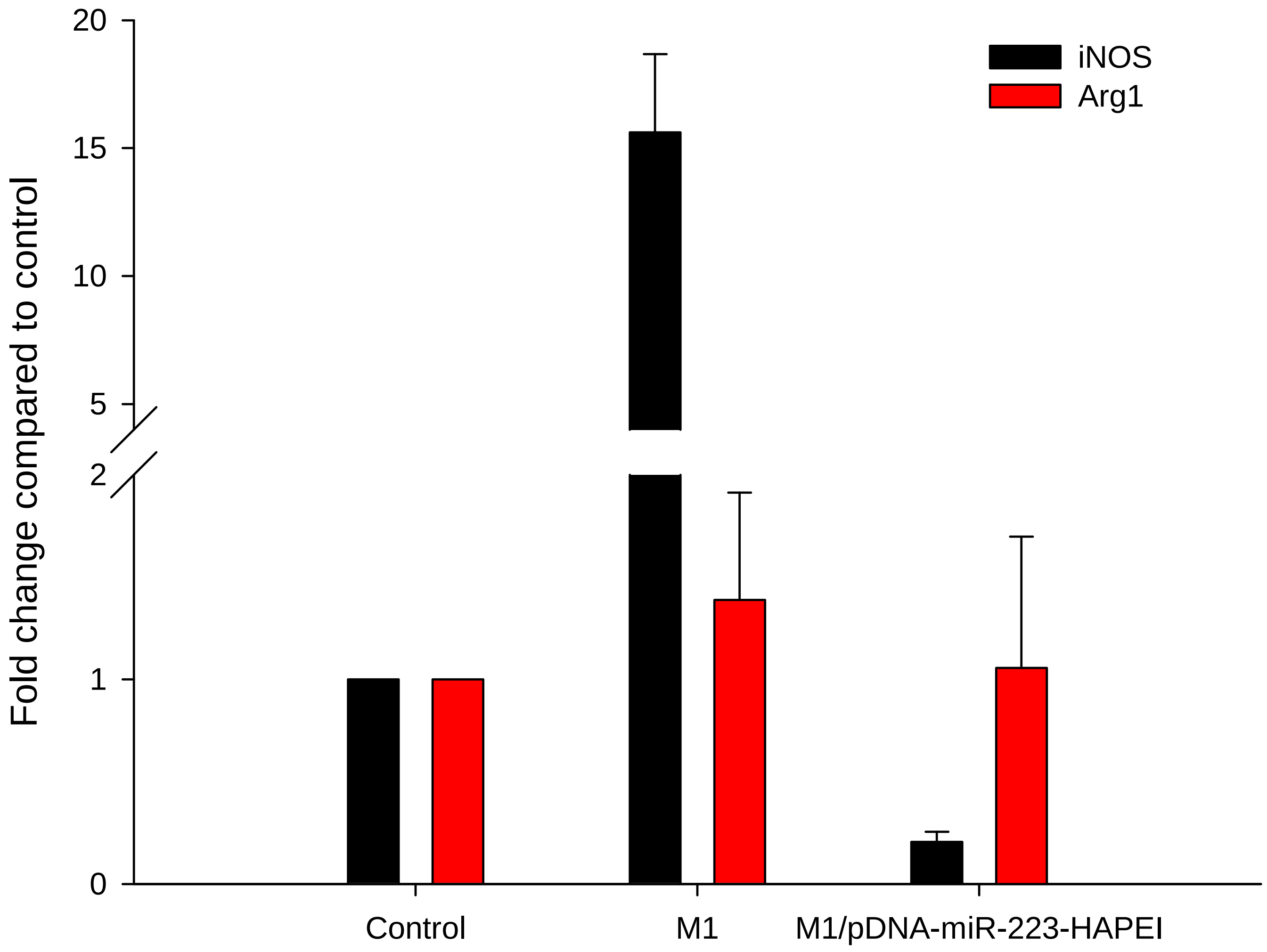

Supplement: S1 Data — (PDF) [file pone.0152024.s001.pdf]
